# Metabolic reprogramming in cancer and senescence

**DOI:** 10.1002/mco2.70055

**Published:** 2025-03-04

**Authors:** Yuzhu Zhang, Jiaxi Tang, Can Jiang, Hanxi Yi, Shu Guang, Gang Yin, Maonan Wang

**Affiliations:** ^1^ Department of Pathology Xiangya Hospital School of Basic Medical Sciences Central South University Changsha China; ^2^ National Clinical Research Center for Geriatric Disorders Xiangya Hospital Central South University Changsha China

**Keywords:** aging, metabolic reprogramming, mitochondrial dysfunction, signaling pathways, tumor

## Abstract

The rising trend in global cancer incidence has caused widespread concern, one of the main reasons being the aging of the global population. Statistical data show that cancer incidence and mortality rates show a clear upward trend with age. Although there is a commonality between dysregulated nutrient sensing, which is one of the main features of aging, and metabolic reprogramming of tumor cells, the specific regulatory relationship is not clear. This manuscript intends to comprehensively analyze the relationship between senescence and tumor metabolic reprogramming; as well as reveal the impact of key factors leading to cellular senescence on tumorigenesis. In addition, this review summarizes the current intervention strategies targeting nutrient sensing pathways, as well as the clinical cases of treating tumors targeting the characteristics of senescence with the existing nanodelivery research strategies. Finally, it also suggests sensible dietary habits for those who wish to combat aging. In conclusion, this review attempts to sort out the link between aging and metabolism and provide new ideas for cancer treatment.

## Graphical abstract

Cancer incidence and mortality rates increase significantly with age, and this rising trend seems to indicate a strong link between aging and cancer. However, despite the commonality between nutrient‐sensing disorders, which are a feature of aging, and metabolic reprogramming, which is a feature of tumors, the specific regulatory relationship between the two is not clear. Organismal metabolism includes gluconeogenesis, lipid metabolism, protein/amino acid metabolism, nucleotide/nucleic acid metabolism, and other nutrient metabolism. Among them, glucose metabolism, lipid metabolism, and amino acid metabolism are the main modes of energy supply for tumor cells, and they are also altered in senescent cells. Therefore, this review will focus on these three modes of metabolism in order to provide us with a deeper understanding of the metabolic associations between aging and tumors. It will also summarize drugs that have been used in clinical trials to target aging and tumor signaling pathways, as well as targeting materials that are in the research phase, and conclude with appropriate dietary recommendations. This will provide new perspectives and ideas for antiaging and tumor prevention research

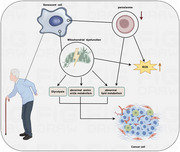

## 1 INTRODUCTION

The aging of the population with cancer has led to continued speculation about the link between cancer and aging. Aging is an unavoidable part of the human life cycle, characterized by a random accumulation of harmful events over time, leading to deterioration of the overall condition of the organism, increased risk of disease and death, morphological changes, functional deterioration, and metabolic dysregulation, resulting in reduced adaptability to the external environment. As individuals age, they often experience a variety of transformations such as genetic instability, the depletion of telomeres, epigenetic reprogramming, loss of proteostasis, a decrease in efficient macroautophagy, faulty nutrient detection, mitochondrial breakdown, the senescence of cells, a reduction in stem cell populations, changes in the communication between cells, persistent inflammation, and disturbances in their internal biological ecosystems.^1^, ^2^ Of these, features such as genomic instability, epigenetic reprogramming, and loss of proteostasis are already recognized as factors that can increase the risk of tumorigenesis, which seems to imply that there appears to be some link between aging and cancer.^3^, ^4^, ^5^, ^6^ Demographic findings clearly indicate that the prevalence and severity of numerous types of cancer rise as individuals grow older; around two‐thirds of newly diagnosed tumors occur in individuals aged 65 years and over.^7^, ^8^, ^9^ Exploring the correlation between aging and tumors is therefore crucial to unraveling the causes of tumors.

Cancer cells cause death through aging, leaving us with the subjective belief that aging inhibits cancer development.^10^, ^11^ However, recent findings indicate that the aging process can trigger the activation of a senescence‐associated secretory phenotype (SASP) and deliver signaling molecules to cancer cells in the form of paracrine secretion to avoid apoptosis, stimulate cancer cell invasion and metastasis, as well as inhibit tumor immunity.^12^ As research delves deeper, it has emerged that aging plays a bidirectional and complex role in tumor development, of which the relationship with tumor metabolic reprogramming is still unclear. Therefore, exploring the correlation between aging and the metabolic changes in tumors is crucial to uncovering the connection between aging and the onset of cancer.

Tumor cells can optimize energy supply through metabolic reprogramming, which promotes swift proliferation, invasion, and the metastasis of cancer cells, thereby accelerating the development of tumors. The main characteristics of tumor metabolic reprogramming are glycolysis, protein decomposition, lipid metabolism, mitochondrial biogenesis, pentose phosphate pathway, and other biosynthesis and bioenergy pathway upregulation. Current studies mainly focus on abnormal glucose metabolism, lipid metabolism, and amino acid metabolism. Carbohydrate is the most important energy supply substance in the cell, and the energy metabolism of tumor cells is mainly glucose metabolism. The main way of glucose metabolism in tumor cells is glycolysis. In other words, glycolytic within tumors is a critical component in the wider framework of cancer metabolic reprogramming. As early as 1924, German scientist WARBURG proposed the concept of “aerobic glycolysis” of tumor cells, that is, compared with differentiated cells, rapidly multiplying cancer cells will deplete a grand amount of glucose to carry out glycolysis and nonaerobic oxidation, even under the condition of sufficient oxygen and generate substantial quantities of lactic acid.^13^, ^14^ The rapidly multiplying cancer cells will expenditure a great amount of glucose for glycolysis instead of aerobic oxidation and produce a substantial quantity of lactic acid. The efficiency of aerobic glycolysis to produce adenosine triphosphate (ATP) is very low in normal cells, but tumor cells impair aerobic respiration in the presence of oxygen through a series of molecular mechanisms to carry out highly efficient glycolysis reaction, which results in a large amount of ATP,^15^, ^16^, ^17^ as well as creating a microenvironment suitable for the survival of tumor cells, creating a proliferative advantage for tumor cells and enhancing their invasive and metastatic abilities.^18^, ^19^ The high flux of glycolysis not only provides energy rapidly but also supplies the essential elements for constructing a wide array of biological molecules in tumor cells.^20^, ^21^ For example, tumor cells are able to synthesize a mix of biomolecules by increasing glucose transporter proteins^22^, ^23^, ^24^ or various key enzymes^25^, ^26^, ^27^ to obtain a more efficient glycolytic reaction to promote metabolism. Second, lipids are a key component of cell membranes and other cellular organelles. Lipids maintain the fluidity of cell membranes and are important for the activation of molecules involved in plasma membrane signaling. Most tumor cells have the ability of de novo synthesis of fatty acids to continuously synthesize new membrane structures. In addition, the active proliferation of tumors requires a constant supply of amino acids for the synthesis of structural and functional proteins. The high metabolic demands of cancer cells and many biochemical reactions require large amounts of amino acids. Studies show that many cancer cells are amino acid dependent, which means that cancer cells lose the ability to synthesize certain nonessential amino acids and require additional supplies.

The occurrence of metabolic abnormalities is complementary to the change of signaling pathway. In addition, disruption of anabolic signaling reduces the organism's nutrition, and reduced nutrition‐sensing signals seem to prolong lifespan and reduce cancer progression. However, common sense suggests that the body must be well nourished in order to have a strong immunity against cancer and prolong lifespan, and the two seem to be contradictory. Based on the above analysis, this paper intends to comprehensively analyze the relationship between aging and tumor metabolic reprogramming by summarizing the relationship between aging and tumor, as well as the similarities and differences between tumor and senescent cell metabolism, and explore the detailed relationship of its molecular mechanism. Meanwhile, this review analyses one of the key factors contributing to cellular senescence, namely, mitochondrial dysfunction, and analyses its relationship with tumorigenesis; since lipid metabolism occurs mainly in peroxisomes, we analyzed its effects on aging and tumor. Also, this review summarizes the current intervention strategies targeting the nutrient sensing pathway and the clinical cases of treating tumors for senescence features and tries to find the tumor treatment strategies based on targeting the senescence pathway. Finally, it also suggests rational dietary habits for people who want to fight aging. In conclusion, this review attempts to sort out the link between aging and metabolism and provide new ideas for cancer treatment.

## 2 THE LINK BETWEEN AGING AND TUMORS

**FIGURE 1:**
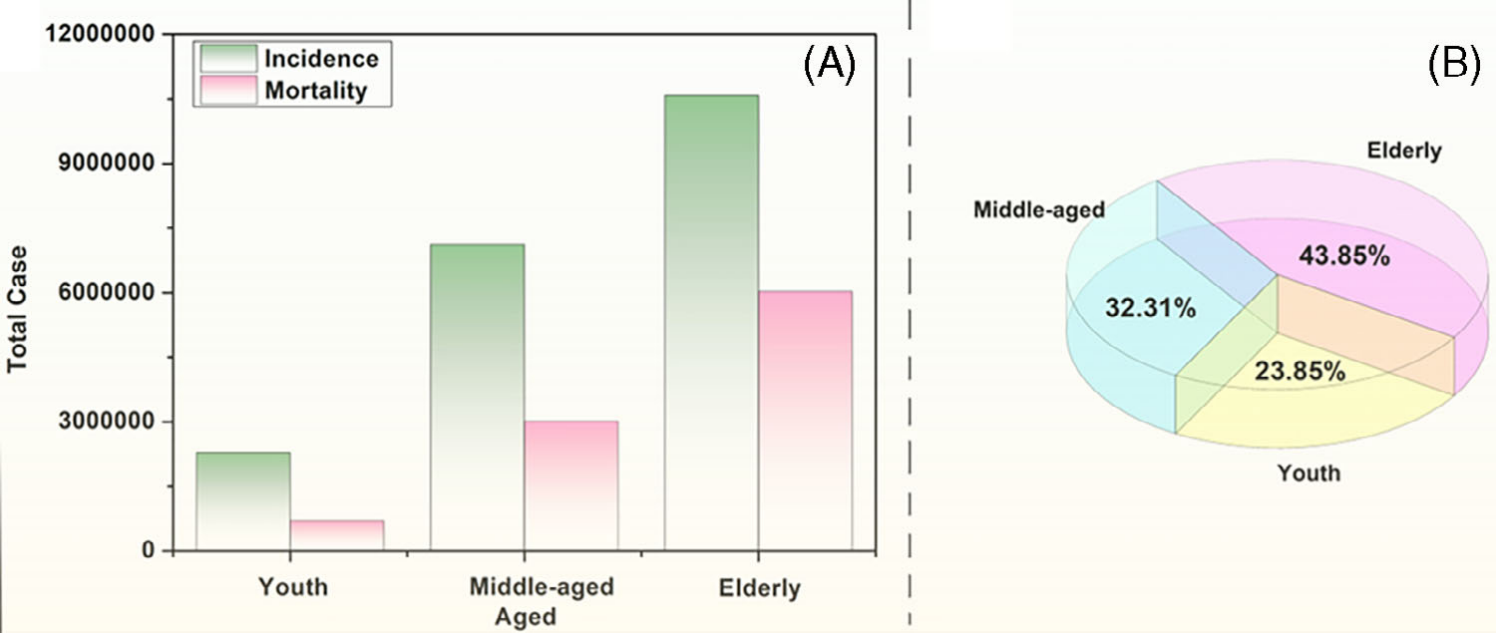
Cancer epidemiological statistics. (A) Statistical map of cancer incidence and mortality across various age groups worldwide. (B) Cancer deaths in different age groups as a percentage of incidence. This figure is drawn by the Figdraw online platform, thanks to Figdraw (https://www.figdraw. com/static/index.html#/).

It appears that the incidence and malignancy of many tumors increases with age. The latest data from Globocan 2022 can further visualize that there is an inevitable link between aging and cancer: from Figure 1A we can clearly see that the probability of developing tumors in humans increases significantly with age, with the incidence rates of cancer in the young, middle‐aged, and elderly populations being 11.4, 35.6, and 53%, respectively; and at the same time, the mortality rate is also gradually climbing, with the number of deaths accounting for 57% of the incidence in the elderly ranking first in the pie diagram in Figure 1B. From the pie chart in Figure 1B, we can see that the number of cancer deaths among the elderly accounted for 57% of the number of incidence cases, ranking the first. This shows that the aging of the organism leads to a gradual decline in diverse body functions and a substantial rise in the occurrence of cancer, implying that aging can promote cancer.

**FIGURE 2:**
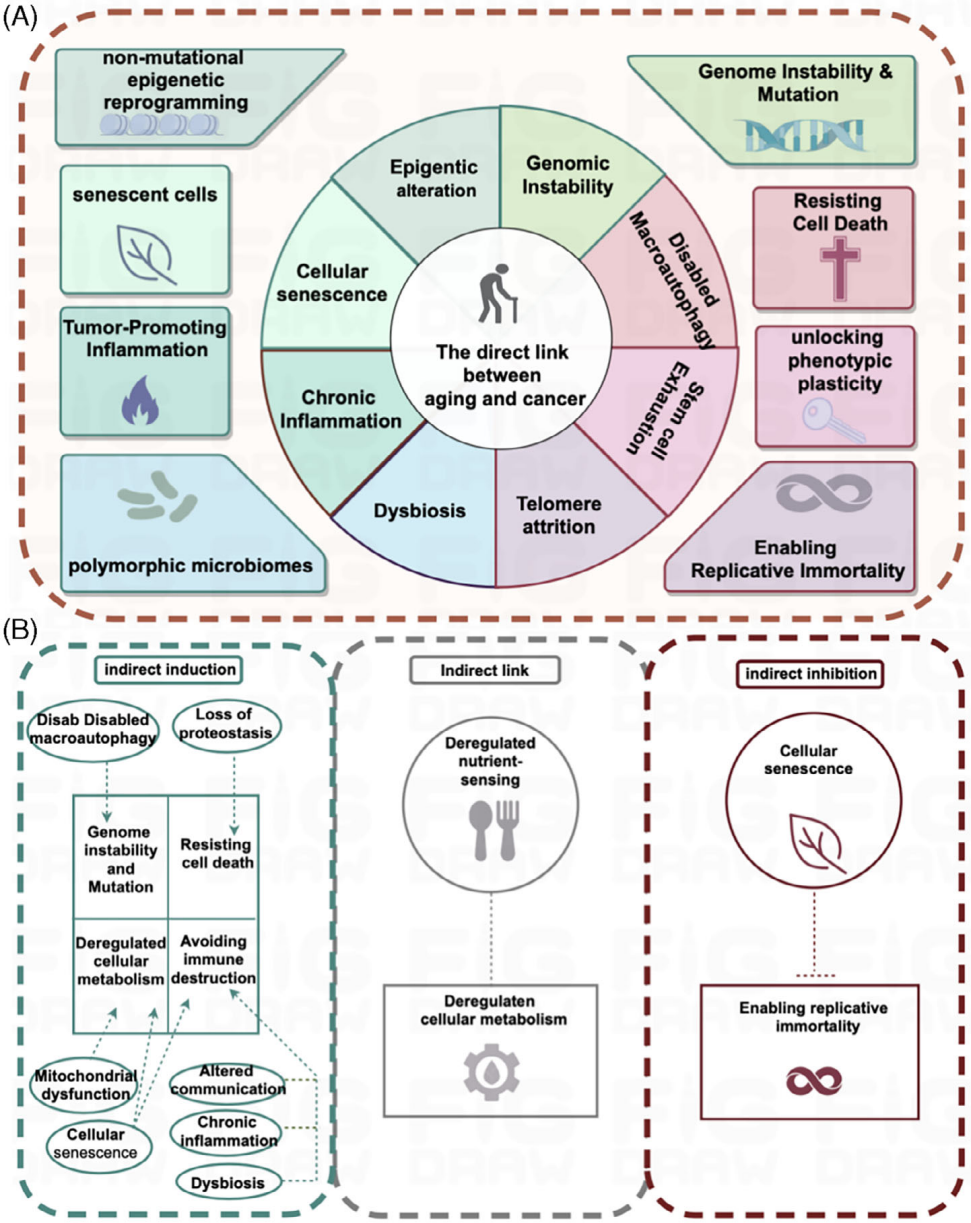
The link between aging and cancer. (A) Direct link. Senescence traits (genetic instability, epigenetic alterations, persistent inflammation, ecological dysregulation, cellular senescence) directly promote tumor traits (genomic instability and mutations, epigenetic reprogramming without mutations, inflammation‐promoted tumors, polymorphic microbiota, senescent cells); senescence traits (telomere shortening, stem cell depletion, mega‐autophagy malfunctioning) directly inhibit tumor traits (ability to replicate indefinitely, phenotypic plasticity, resistance to cell death). (B)Indirect link. Senescence features (macroautophagy dysfunction, loss of proteostasis, mitochondrial dysfunction, cellular senescence, altered intercellular communication, chronic inflammation, ecological dysregulation) may indirectly induce tumor features (genomic instability and mutation, cellular resistance to death, metabolic reprogramming, immune escape); senescence features (cellular senescence) may indirectly inhibit tumor features (ability to replicate indefinitely); and there may be an association between senescence features (nutrient sensing dysregulation) and tumor traits (metabolic reprogramming) may be associated. This figure is drawn by the Figdraw online platform, thanks to Figdraw (https://www.figdraw. com/static/index.html#/).

To explore the link between aging and tumors, we need to understand the characteristics of each and whether there is a connection between these characteristics. Of the 12 features of aging, although three features such as loss of proteostasis, mitochondrial dysfunction, and altered intercellular communication^28^, ^29^ lack clear counterparts in cancer and the relationship between aging‐associated nutrient sensing dysregulation and cancer‐associated cellular metabolic reprogramming was unclear and required specific analysis for specific forms of metabolism, the remaining eight features, which form a direct correspondence with those of cancer (see Figure 2A), are: features common to both aging and cancer (genomic instability, epigenetic alterations, chronic inflammation, and environmental imbalance.); aging‐antagonistic characteristic, that is, telomere shortening and stem cell weakening, that prohibit the emergence of certain features of cancer (capacity for infinite replication and phenotypic plasticity); and under specific circumstances, macroautophagy dysfunction inhibits the onset of cancer features (resistance to cell death), and the senescence program can deliver signaling molecules to cancer cells via SASP to avoid apoptosis.

Based on the relationship between senescence features and tumors, these eight features of senescence can be^1^, ^25^, ^30^, ^31^ divided into three categories: one category of features and those of cancer are mutually inhibitory, for example, telomere depletion induces permanent cell cycle arrest and destroys the feature of infinite replication of tumor cells; stem cell depletion leads to the loss of cellular plasticity required for tissue repair, and the continued differentiation of tumor cells hinders the sustained proliferation required for tumors.^12^ The second category of features promotes tumors, such as genomic instability where DNA is damaged by endogenous and exogenous stimuli, and with aging this damage intensifies, resulting in altered activity of pertinent genes. This culminates in the development of cancer and epigenetic reprogramming causing extensive changes in the epigenome,^32^, ^33^, ^34^ which favors changes in the ability to select features for phenotypic selection, can lead to clonal growth of tumor cells, enhancing proliferation and expansion; along with the aging process, the body usually experiences chronic inflammation, and immune cells promote tumor progression, by providing bioactive molecules to the tumor microenvironment.^35^, ^36^, ^37^ Inflammation can promote a wide array of tumor signature functions; a good gut microbiota is important for maintaining human health, and aging of the body can easily lead to the destruction of the normal flora structure,^38^ which causes ecological dysregulation leading to tumorigenesis. The third group of features refers to macroautophagy disorders and cellular senescence, which have a dual effect on tumorigenesis. It has been found that the articulation of genes related to autophagy ATG5, ATG7, and BECN1 decreases with age.^39^ Knockdown of ATG5^40^, ^41^ and ATG7^42^ significantly accelerates cell‐autonomous senescence, and a transient inhibition of autophagy can significantly promote tumorigenesis, suggesting that autophagy can play an oncostatic role by inhibiting the property of tumor cells to resist cell death; overexpression of ATG5 transgene^43^ or BECN1^44^, ^45^ gain‐of‐function mutation can stimulate autophagy, delay senescence, and reduce the incidence of tumors.

In addition to the direct links, some features of senescence lack clear counterparts in cancer, but they may play an indirect role in inducing or inhibiting specific features of cancer, as shown in Figure 2B. Macroautophagy dysfunction may induce the onset of genomic instability and mutations in cancer features; loss of proteostasis may favor cancer features to resist cell death; mitochondrial dysfunction and cellular senescence may induce metabolic reprogramming of cancer trait cells; cellular senescence, altered intercellular communication, chronic inflammation, and ecological dysregulation may induce tumor cells to undergo immune escape. Cellular senescence may block the mechanism of infinite replication of tumor cells and inhibit cancer proliferation. What is more, the relationship between senescence‐associated nutrient sensing dysregulation and cancer‐associated cellular metabolic reprogramming remains unclear and needs to be analyzed specifically for specific metabolisms. Body metabolism includes glucose metabolism, lipid metabolism, protein/amino acid metabolism, nucleotide/nucleic acid metabolism, and other nutrient metabolism. Various substance metabolisms are interconnected and interact with each other, and metabolic signaling pathways interact to form a complex metabolic network. The metabolic pattern of tumor cells is very different from that of normal cells. Tumor cells reprogram their metabolic pattern, which contributes to their rapid growth. The main features of metabolic reprogramming in tumor cells include upregulation of glycolysis, amino acid metabolism, lipid metabolism, mitochondrial biogenesis, pentose phosphate pathway, and other biosynthetic and bioenergetic pathways. Among them, glycolysis, lipid metabolism, and amino acid metabolism are the main energy supply modes in tumor cells, which are also altered in senescent cells. In summary, in this section, we have discussed the relationship between aging and tumors, and we will next focus on a more in‐depth discussion of the links between aging and glucose metabolism, lipid metabolism, and amino acid metabolism in tumors.

## 3 AGING PROMOTES METABOLIC REPROGRAMMING IN TUMOR CELLS

Tumor cell metabolic reprogramming is a key feature in tumor biology that involves significant changes in multiple metabolic pathways to accommodate the rapid proliferation of tumor cells. Glycolysis is one of the most prominent features of tumor cell metabolic reprogramming. While this modality is not as high as oxidative phosphorylation in terms of net ATP production, cancer cells compensate for this deficiency by promoting higher rates of glycolysis through increased glucose uptake. Second, lipid metabolism is also a key component of metabolic reprogramming in tumor cells. Tumor cells meet the biosynthetic demands of the cell membrane by enhancing lipid synthesis, which is important for rapid tumor cell proliferation and migration. Amino acid metabolism is also significantly altered in tumor cells. In particular, glutamine metabolism is upregulated in tumor cells to support the biosynthetic needs and energy production of tumor cells. Next, we will delve into the interconnections of glucose metabolism, lipid metabolism, and amino acid metabolism in aging and tumor processes. This part of the study will focus on revealing how these metabolic pathways act and influence cellular function in aging and tumor development. We will analyze in detail how these metabolic changes contribute to tumor cell progression and the effects of aging on the three metabolic patterns in tumor cells. Through this in‐depth analysis, we can better understand the complex metabolic relationship between aging and tumors and provide potential targets for future therapeutic strategies.

### 3.1 Glycolytic reprogramming in tumors

**FIGURE 3:**
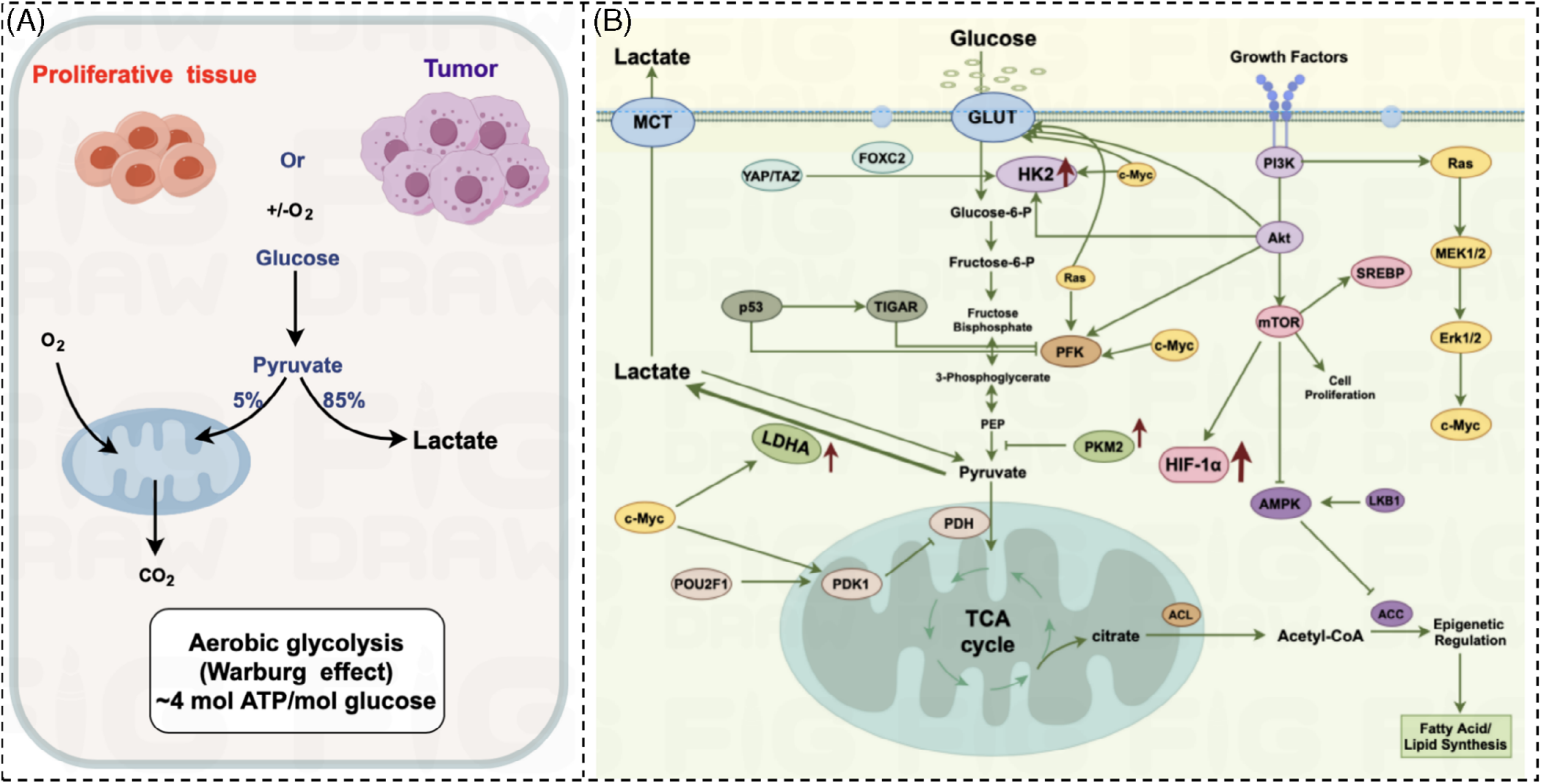
Aerobic glycolysis in tumor cells. (A) Warburg effect. Cancer cells exhibit a preference for metabolizing glucose to break down pyruvate, even when oxygen is plentiful; a large amount of pyruvate is converted to lactate, that is, aerobic glycolysis; a minimal fraction of pyruvate is transported into the mitochondria for catabolic processes that result in the formation of CO_2_. Through the course of aerobic glycolysis, 4 mol of ATP is generated from the consumption of 1 mol of glucose. (B) Diagram of aerobic glycolysis signaling pathway in tumor cells. Aerobic glycolysis in tumor cells promotes high levels of HIF‐1 expression and activates multiple pro‐oncogenic signaling pathways; the activation of these signaling pathways upregulates the transcription levels of the pivotal glycolytic enzymes, hexokinase 2, and lactate dehydrogenase A, which in turn promote aerobic glycolysis in tumor cells. This figure is drawn by the Figdraw online platform, thanks to Figdraw (https://www.figdraw. com/static/index.html#/).

We know from the previous section that the relationship between aging‐associated nutrient sensing dysregulation and cancer‐associated cellular metabolic reprogramming remains unclear and needs to be analyzed specifically for specific metabolisms. In this paper, we will focus on the reprogramming of glucose metabolism. Glucose is the most important energy‐supplying substance in the organism, and its metabolic pathway is mainly divided into glycolysis pathway, pentose phosphate pathway, and oxidative phosphorylation. Normal cells in aerobic conditions, mainly through oxidative phosphorylation to produce ATP; in anaerobic conditions, mainly through glycolysis to produce ATP, while the swift multiplication of cancer cells necessitates an escalated energy requirement during the growth process, leading to the process of reprogramming the metabolism of tumor cells. In 1924, the concept of aerobic glycolysis in cancer cells was first introduced by German scientist, WARBURG.^46^, ^47^ Compared with differentiated cells, tumor cells undergoing rapid proliferation tend to use significant amounts of glucose for glycolytic instead of aerobic oxidation, subsequently generating substantial quantities of lactic acid even when there is ample oxygen available (Figure 3A). Cancer cells consume glucose to produce pyruvate either aerobically or anaerobically. 85% of pyruvate undergoes glycolytic to produce lactate; only a small amount of pyruvate undergoes oxidative phosphorylation aerobically to produce CO_2_. Normal cells aerobically glycolyzes ATP very inefficiently, but in tumor cells, glycolysis is 20–30 times more efficient than in normal cells.^48^ The acceleration of glycolysis has been observed to have a positive correlation with the rapid expansion of tumor cells,^15^, ^49^ indicating a marked association with the aggressive advancement of malignant tumors.^18^, ^50^ This intricate relationship underscores the fundamental role played by glycolytic pathways in fueling the heightened energy demands characteristic of proliferating cancerous cells. The promotion of glycolysis not only provides a metabolic advantage to cancer cells but also contributes significantly to the metastatic potential and invasiveness exhibited by malignant neoplasms.

Although glycolysis capacity is less efficient compared with mitochondrial oxidative phosphorylation, aerobic glycolysis offers numerous advantages to the proliferation of cancer cells: (1) aerobic glycolysis produces ATP extremely fast,^20^, ^50^ which can immediately supply energy to rapidly proliferating tumor cells; (2) tumor cells can obtain a large amount of intermediate metabolites through aerobic glycolysis,^20^, ^51^ which are used to synthesize fats, proteins and nucleic acids to meet their active anabolic demands; (3) the process of aerobic glycolysis within tumor cells generates a high volume of lactic acid. This in turn causes an increase in acidity within the tumor's microenvironment.^52^, ^53^ This acidic shift has been seen to promote angiogenesis within tumor cells, enhance their invasiveness, and facilitate metastasis and evasion from immune responses; (4) the aerobic glycolysis in tumor cells has the ability to facilitate the expression of the hypoxia‐inducible factor‐1 (HIF‐1).^54^, ^55^, ^56^ This occurs through the molecular process wherein mTOR, a target downstream of phosphatidylinositol 3‐kinase (PI3K)/Akt in tumor cells, plays a role in the transcriptional regulation of HIF‐1. Consequently, this leads to an elevated level of HIF‐1 expression within the tumor cells,^57^ which results in high levels of HIF‐1 expression, which in turn increases the manifestation of glycolytic enzymes such as hexokinase 2 (HK‐2), M2‐type pyruvate kinase (PKM2), and lactate dehydrogenase A (LDHA), along with the regulatory enzyme pyruvate dehydrogenase kinase isozyme 1 (PDK1).^58^, ^59^ The signaling pathways involved are shown in Figure 3B and are summarized below:

(1) PI3K–AKT–mTOR pathway^60^: PI3K activates AKT, which activates HK2, phosphorylates PFK, and activates mTOR to stimulate glycolysis.
(2) Hippo pathway^61^, ^62^: Activation of the Hippo pathway phosphorylates YAP/TAZ and induces HK2 expression via FOXC2, promoting glycolysis.
(3) Myc signaling pathway^63^, ^64^: Myc induces the glycolytic enzymes HK2 and PFK, which catalyze the corresponding glycolytic steps and promote glycolysis.
(4) LKB1/AMP‐activated protein kinase (AMPK) signaling pathway^65^: LKB1 upregulates the expression of AMPK, a key target of AMPK, acetyl coenzyme A carboxylase (ACC), which this process facilitates the transformation of acetyl coenzyme A into malonyl coenzyme A.^66^, ^67^ AMPK inhibits ACC activity and promotes the level of intracellular glycolysis occurring.
(5) Ras signaling pathway^68^, ^69^: Upon stimulation by growth factors, the oncogene Ras is triggered into action. It interacts with the MEK protein, leading to the activation of MEK.^68^, ^70^ Subsequently, MEK in turn triggers the downstream substrate, ERK. This cascade effect ultimately boosts the expression of c‐Myc, thereby facilitating glycolysis.
(6) p53 signaling pathway: p53^71^ is able to transcriptionally activate SCO2^72^, ^73^ and other mitochondrial electron transport chain proteins to promote intracellular mitochondrial activity.

These are the signaling pathways involved in tumor aerobic glycolysis that have been reported so far.

### 3.2 Aerobic glycolysis in senescent cells

Senescent cells are characterized by morphological and metabolic changes, chromatin remodeling, altered gene expression, as well as the emergence of a proinflammatory phenotype known as SASP and a number of senescence‐related markers, summarized as follows: (1) β‐Galactosidase Staining Kit,^74^ the increase in its activity reflects the increase in lysosomal content of senescent cells,^75^ and is the most commonly used senescence marker. (2) p53^76^ is a key regulator of cell cycle regulation, and its activation can lead to cell cycle arrest, thus triggering cellular senescence; phosphorylated p53^77^ is a commonly used marker of cellular senescence. activation of the DNA damage response can lead to the phosphorylation of p53, thus leading to cellular senescence. (3) p21 is a key regulator of cell cycle regulation,^75^ and its upregulation is one of the important markers of cellular senescence. p21 can inhibit the activity of CDK,^78^ leading to cell cycle arrest, thus triggering cellular senescence. (4) p16 can directly inhibit the activity of CDK4/6,^79^ which leads to cell cycle arrest and triggers cells to enter senescence. (5) LaminB1 is a structural protein of the nuclear membrane,^80^ and its decreased expression is an important hallmark of cellular senescence. Downregulation of LaminB1 leads to heterochromatin decondensation and the production of cytoplasmic chromatin fragments, which cause DNA damage.^75^, ^81^ The downregulation of LaminB1 mRNA in transcriptomic studies is a widespread hallmark of cellular senescence. The general downregulation of LaminB1 mRNA in transcriptomics studies is a widespread hallmark of cellular senescence. (6) SASP includes increased secretion of extracellular matrix metalloproteinases, chemokines, cytokines and growth factors, and so on.^75^ SASP is an important feature of cellular senescence, and its regulatory mechanism involves endoplasmic reticulum stress,^82^ cellular membrane composition, and other dimensions, which is a complex biological process. (7) When the cell is stimulated, Rb is phosphorylated^83^ and thus loses its inhibitory effect, causing the cell cycle to enter the G1/S checkpoint and ultimately leading the cell to enter the senescent state. (8) γ‐H2A.X^84^ is one of the commonly used markers of cellular senescence and is an indicator of the DNA damage response. (9) 53BP1^85^ plays an important role in DNA repair by binding to p53 and enhancing its transcriptional activity. But whether 53BP1 stays or not depends on γ‐H2A.X. (10) Ki67 is a tumor cell proliferation marker that is not detected when cells are in the G0 resting phase. Senescent cells, on the other hand, are marked by a permanent exit from the cell cycle and therefore senescent cells do not express Ki67.^86^

**FIGURE 4:**
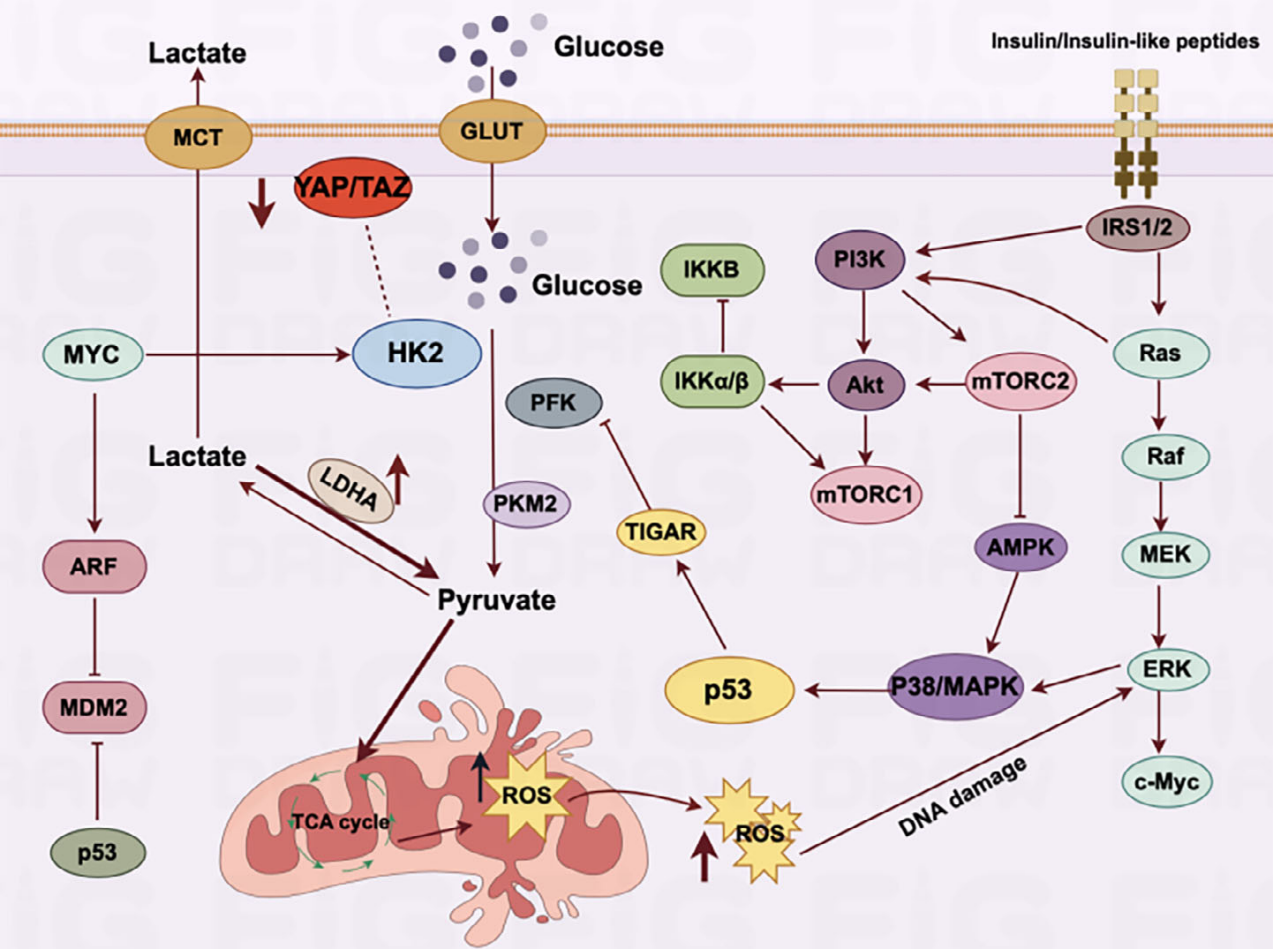
Diagram of glycolytic signaling pathway in senescent cells. The massive oxidation of lactate to generate pyruvate in senescent cells promotes oxidative phosphorylation to generate large amounts of ROS, causing intracellular DNA damage. This figure is drawn by the Figdraw online platform, thanks to Figdraw (https://www.figdraw. com/static/index.html#/).

In addition to these markers, glycolysis also occurs in senescent cells, and the specific pathways are shown in Figure 4.This metabolic shift is evidenced by an upregulation of glycolytic enzymes and glucose transporter proteins, particularly GLUTs, enabling these cells to favor the glycolytic pathway for energy production. The biochemical processes of the TCA cycle and oxidative phosphorylation activity are not just fundamental to cell physiology, their nuances are proving instrumental in understanding the unique mechanisms of cellular energy production and consumption. In recent explorations, it has been observed that the TCA cycle and oxidative phosphorylation—the principal engines of energy production in cells—both exhibit increased activity,^87^, ^88^, ^89^ which is somewhat different from tumor cells. In tumor cells, aerobic glycolysis is preferred, and mitochondrial respiration is relatively weakened. The activation and enhanced function of HIF‐1α in malignant tumor cells precipitate a notable escalation in the levels of numerous critical glycolytic enzymes, including but not limited to HK‐2, PKM2, LDHA and the pivotal regulatory enzyme PDK1.^58^, ^90^ The heightened activity of these enzymes results in elevated intracellular aerobic glycolysis, higher lactate levels, and a decrease in mitochondrial oxidative phosphorylation. In aging cells, aside from using glycolysis for energy, there is a significant oxidation of lactate to produce pyruvate to enhance mitochondrial respiration, unlike in actively dividing cells, which primarily depend on glycolysis for rapid energy production. Senescent cells show a marked reliance on the mitochondria to generate ATP through the oxidative phosphorylation pathway,^91^, ^92^, ^93^ which in turn generates a large amount of reactive oxygen species (ROS).

### 3.3 Senescence promotes aerobic glycolysis in tumor cells

**FIGURE 5:**
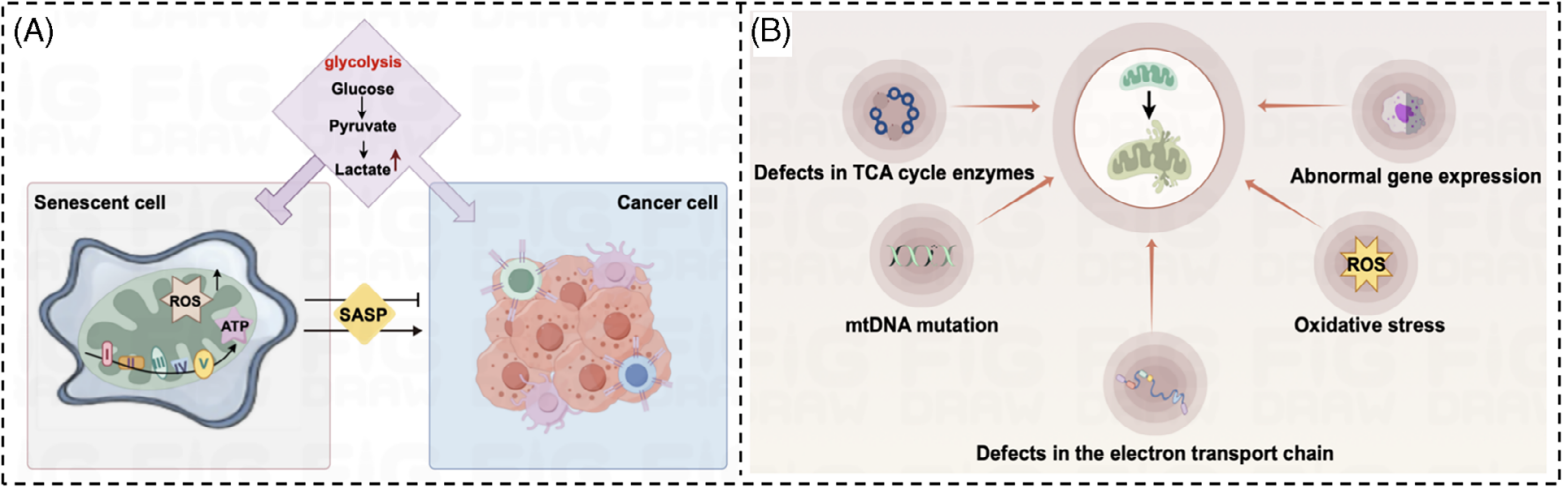
Aging and tumors and one of the characteristics of aging: the effect of mitochondrial dysfunction on tumorigenesis. (A) Relationship between senescence, glycolysis, and tumors. Aging cells exhibit a distinct high metabolic phenotype characterized by enhanced glycolysis, the TCA cycle, and oxidative phosphorylation. Glycolysis inhibits senescence but promotes malignant tumor progression; senescence can promote malignant tumor progression through glycolysis. The SASP fosters the onset of cellular aging while simultaneously acting as a deterrent to the development of cancer on the one hand. On the other hand, SASP also promotes tumorigenesis by stimulating inflammation‐related responses. (B) Mitochondrial dysfunction. The causes of mitochondrial dysfunction are defective TCA cycle enzymes, oxidative stress, alterations in the mitochondrial DNA (mtDNA), impairments within the electron transport chain, and abnormal gene expression. This figure is drawn by the Figdraw online platform, thanks to Figdraw (https://www.figdraw. com/static/index.html#/).

Glycolytic signaling pathways in tumor and senescent cells were elaborated separately, and we found that there seems to be some connection between senescence, tumor, and glycolysis. Current research seems to tell us that glycolysis inhibits cellular senescence. For example, Korolchuk et al.^94^ found that knocking out the mitochondria of human embryonic lung MRC5 fibroblasts stopped cellular senescence, which was due to the fact that the destruction of mitochondria led to an increase in glycolysis, resulting in the production of an abundance of lactic acid, which in turn causes cells to escape aging, which allowed the cells to escape from senescence. Flores et al.^95^ found that hair follicle stem cells normally break down glucose into pyruvate, which in turn generates lactate via the glycolytic pathway. If lactate production is blocked, the activation of hair follicle stem cells can be significantly inhibited; on the other hand, in a mouse model, lactate production within hair follicle stem cells is a key factor in promoting hair growth. When the secretion of lactate within hair follicle stem cells is amplified, it leads to a substantial acceleration in hair growth in mice. This phenomenon underscores the pivotal role played by lactate in regulating the proliferation and differentiation of hair follicle stem cells. This amplification in hair growth was attributed to the increased energy supply and metabolic reprogramming that lactate facilitated within the hair follicle stem cells.^96^, ^97^, ^98^ Li et al.^99^ found that when expression levels of the glycolytic key enzyme PKM2 are elevated, it can significantly diminish the aging of vascular endothelial cells.^100^, ^101^ These results undoubtedly indicate that glycolysis is a key enzyme in the growth of hair. These results are undoubtedly not suggesting that glycolysis can inhibit cellular senescence (Figure 5). Nevertheless, the preponderance of research within this domain has predominantly favored nontumorigenic cells, with only a scant number of studies meticulously probing the intricate relationship between glycolysis and cellular senescence within the confines of cancer cells. The significant paucity in scientific research has created a substantial lacuna in our comprehension of the mechanisms through which cancer cells modulate the metabolic pathways that undergo fundamental alterations during the aging process. This discrepancy in investigation not only hampers our understanding of the intricacies of cancer metabolism but also obstructs the potential development of effective therapeutic strategies targeting metabolic dysregulation in aging‐related cancers. The dearths of insights into the synergistic interplay between the glycolytic profile and the aging mechanisms of tumor cells remain a conspicuous void, awaiting comprehensive exploration to elucidate the full spectrum of implications for cancer progression and potential therapeutic interventions.

The marked increase in tumor incidence with age has focused our attention on the cancer‐promoting effects of aging. From a metabolic perspective, we know that tumor cells can alter energy metabolic pathways through metabolic reprogramming to accommodate their abnormal growth and reproduction needs. Senescent cells have the ability to spontaneously change their metabolism by producing an abundance of protein factors, known as SASPs^102^, which can impact the functioning of surrounding cells within the organism's microenvironment.^103^ This process can be likened to the way glucose affects the body. Glucose, serving as the primary energy source for aging cells, undergoes metabolic transformations as well, with changes in the intensity of glycolysis in senescent cells^104^, ^105^ and TCA cycling activity of senescent cells^106^ are changed. In the metabolic study of senescent cells, researchers found that senescent cells were able to further utilize oxidative phosphorylation metabolites such as citric acid, α‐ketoglutarate fumarate, and malic acid to enhance cellular mitochondrial respiration and generate large amounts of ROS, which ultimately induced other cellular senescence.^107^, ^108^, ^109^ Concurrently, senescent cells exhibit an increase in metabolites closely associated with glycolysis, such as lactate^104^ and pyruvate, which stimulate the glycolytic process^107^—a phenomenon reminiscent of the aerobic glycolysis observed in cancer cells. In addition, some genes related to glycolysis and TCA cycle in senescent cells, such as glucose transporter protein 1 (GLUT1),^110^, ^111^ phosphoglycerate isomerase (PGI),^112^ phosphoglycerate kinase 1 (PGK1),^113^ and citrate synthase (CS), were also significantly upregulated. In conclusion, aging cells exhibit a distinct high metabolic phenotype characterized by enhanced glycolysis. The alteration of energy metabolic pathways as observed in the TCA cycle and oxidative phosphorylation is evocative of the metabolic reprogramming that occurs in tumor cells. This metabolic adaptation enables tumor cells to meet their increased energy demands and sustain rapid proliferation, despite the less efficient ATP production associated with glycolysis. Furthermore, the reprogramming of metabolic pathways in tumor cells not only fuels their growth and proliferation but also contributes to the development of an immunosuppressive tumor microenvironment. The dysregulated metabolism of tumor cells not only serves as a hallmark of cancer but also presents potential therapeutic targets to disrupt the energetic advantage of malignant cells (Figure 5A). We therefore speculate that senescence may promote aerobic glycolysis in tumor cells. In the following, we will argue our point of view in the light of currently available studies.

Senescence in tumor cells generates ROS that induce oxidative stress in neighboring tumor‐associated fibroblasts undergoing aerobic glycolysis.^114^, ^115^ Senescence leads to oxidative stress in tumor cells,^116^ and this oxidative stress environment contributes to the shift of tumor cells toward aerobic glycolysis,^117^, ^118^ which promotes tumor development; in addition, in the intricate landscape of cellular biology, senescent cells—the aging or deteriorated cells that have ceased to divide—displayed a notable rise in aerobic glycolysis and the production of lactate that is contingent upon the activity of PDK4.^119^, ^120^ In turn, the large amount of lactate formed generates ROS, which ultimately contributes to the senescence of the surrounding cells and even the tissue system, and promotes tumor proliferation. In summary, although the research on senescence and tumor aerobic glycolysis is not in‐depth, based on the above studies, we believe that senescent cells can spontaneously change their metabolism, just like tumor cells, and that senescence promotes aerobic glycolysis in tumor cells, which in turn promotes tumor malignant progression.

### 3.4 Abnormalities of lipid metabolism in tumor cells

There is a close connection between senescence and tumors, and for tumor treatment, analyzing lipid metabolic reprogramming in senescence and tumors to find the connection between the two will provide a relevant theoretical basis for clinical precision treatment. Next, we will discuss lipid metabolism reprogramming in tumor cells and senescent cells.

In addition to glucose metabolism, lipid metabolism is one of the important metabolic pathways in the body, including triglyceride metabolism, phospholipid metabolism, and cholesterol metabolism. Triglyceride is an important form of energy storage and oxidative energy supply; phospholipids constitute an important phospholipid bilayer of biological membranes and are involved in the transduction of signaling molecules; cholesterol also constitutes biological membranes and is prevalent in bile acids and signaling molecules. Among the many lipids, the most dominant component is fatty acids, the β‐oxidation of which in normal cells provides energy for cellular physiological activities. In the process of tumorigenesis, the abnormal proliferation of tumor cells is a more significant feature of malignant tumors; in the process of abnormal proliferation of tumor cells, the formation of cell membrane and the formation of related signaling molecules inevitably require the participation of lipid metabolism; at the same time, the rapid proliferation of tumor cells require a large amount of energy supply. In addition to glucose metabolism, the majority of the energy supply comes from lipid metabolism. Therefore, the high metabolic requirements of tumor growth lead to the reprogramming of lipid metabolism in tumor cells. In 1953, Medes et al.^121^ experimentally demonstrated an increase in the synthesis of lipids from scratch in tumor cells and suggested that acetate and glucose carbon could be used for fatty acid chain synthesis, but their synthesis rate could not meet the lipid requirements of rapidly growing tumors, and thus tumors must also take up preformed lipids from the host. In 2004, Swinnen et al.^122^ proposed that in prostate cancer androgens orchestrate the stimulation of lipogenic gene expression through a molecular mechanism that regulates the activation of cholesterol regulatory element‐binding protein (SREBP), thereby indirectly controlling increased lipogenesis, and suggested that as tumors progress, increased lipogenesis persists or re‐emerges with the development of androgen‐independent cancers. In 2014, Roy et al.^123^ demonstrated experimentally that HSulf‐1 deletion promotes fatty acid synthesis while inducing lipogenic gene expression to stimulate lipid droplet accumulation, leading to reprogramming of lipid metabolism during ovarian cancer progression and promoting ovarian cancer progression. In addition, relevant literature reports indicate that in addition to the reprogramming of lipid metabolism in tumor cells during tumorigenesis and progression, lipid metabolism reprogramming also occurs in the tumor microenvironment, leading to the accumulation of lipids in the tumor microenvironment, which promotes tumor metastasis as well as immune resistance against tumors.^124^ CD36, an exogenous lipid transporter molecule, has been found to be associated with poor prognosis in many cancers. Upregulation of CD36 expressed on the surface of T cells will lead to the accumulation of lipids, which will inhibit the secretion of antitumor factors, such as IFN‐γ and TNF‐α, and thus increase the immune resistance of tumors.^125^ Based on the evidence from the above studies, we believe that the reprogramming of lipid metabolism in tumor cells mainly involves the processes of increased lipid uptake, increased lipid synthesis, and increased lipid storage, and that the reprogramming of lipid metabolism in tumors will promote tumor development. The process of lipid metabolism regulation in tumors is extremely complex, and the following signaling pathways and targets have been reported to be closely related to the regulation of lipid metabolism in tumors:

1. PI3K–AKT signaling pathway: Activation of mTORC1 through the PI3K/AKT signaling pathway in response to growth factors or activated RSA genes. mTORC1 induces mature SREBP to enter the nucleus, and SREBP promotes transcription of genes related to lipid metabolism, which increases the synthesis of lipids in the tumor cells to meet the metabolic needs of the tumor cells for abnormal proliferation.^126^
2. NF‐κB signaling pathway: Activation of NF‐kB signaling pathway inhibits triglyceride catabolism by inhibiting the genes of triglyceride lipase (ATGL)/Brummer and promotes tumor growth and proliferation by regulating Ces1d/CES1.^127^
3. STAT3 signaling pathway: Activation of STAT3 signaling pathway promotes malignant development and drug resistance of tumors by increasing the expression of the key enzyme for lipid synthesis, FASN, and by activating the actions of the transcriptional regulators SREBPs and peroxisome proliferator‐activated receptors (PPARy).^127^
4. Hippo signaling pathway, which promotes tumor growth and proliferation through the actions of YAP as a coactivator of the transcriptional regulators SREBP‐1c and SREBP‐2 in the nucleus, increasing the expression of genes related to lipid synthesis or directly elevating cholesterol levels. (7)
5. Wnt/β‐catenin signaling pathway, which promotes the formation of lipid‐unsaturated fatty acids and increased synthesis of lipids from scratch by activating the Wnt/β‐catenin signaling pathway, activating SREBPs, and increasing the expression of SCDs.^127^
6. Notch signaling pathway: Notch signaling activation increases the expression of SCDs, promotes the formation of unsaturated fatty acids, and promotes tumor growth and proliferation.^127^

### 3.5 Abnormal lipid metabolism in senescent cells

Senescence is an unavoidable process in organismal cells, and the main features of senescent cells are inability to repair tissues by division, cell cycle arrest, complex SASP in senescent cells, and metabolic reprogramming. Senescent cells have their special lipid metabolism, which is manifested by increased lipid accumulation, and the dysregulation of lipid accumulation in senescent cells may be due to increased lipid uptake, upregulation of lipid biosynthesis pathways, or dysregulation of lipid catabolism.^128^ Relevant literature reports indicate that senescent cells promote lipid uptake through increased expression of CD36, LDLR, and CAV‐1 on the cell membrane; the transcriptional regulator cholesterol regulatory element‐binding protein (SREBP) regulates the increased expression of the key rate‐limiting enzymes (FASN, ACC, and HMGCR) in lipid metabolism, which in turn promotes the synthesis of lipids in senescent cells and promotes the onset and development of senescence; ROS accumulation promotes enhanced fatty acid β‐oxidation and lipid peroxidation in senescent cells^129^

### 3.6 Aging promotes reprogramming of lipid metabolism in tumor cells

Many studies have shown that senescent cells are widely distributed in the tumor microenvironment and that cell cycle arrest and immunogenicity of senescent cells regulate the growth of cancer cells and influence the progression and metastasis of malignant tumors.^129^ In 2021, Yasuda et al.^130^ found that senescent cancer‐associated fibroblasts (CAFs) enhanced gastric cancer (GC) peritoneal tumor formation through the JAK/STAT3 signaling pathway in the tumor microenvironment. According to the previous narrative, senescent CAFs act in the same signaling pathway as lipid metabolism in tumors—the STAT3 signaling pathway, which seems to tell us that there is a relationship between senescence and lipid metabolism reprogramming in tumors. In 2020, Alicea et al.^131^ found that melanoma cells take up lipids from senescent fibroblasts through FATP2 and utilize them to resist targeted therapy. Recent studies have found that in ovarian cancer the senescent lipid microenvironment is more susceptible to ovarian cancer growth by showing that senescence promotes the growth and proliferation of ovarian cancer cells through lipid metabolism.^131^ In addition to this, there is evidence that lipid metabolism in the tumor microenvironment affects T‐cell senescence, which in turn affects immune resistance to tumors.^132^ Although few studies have been reported on the role of lipid metabolic reprogramming of senescent cells on tumors, based on the above studies, it is not difficult to find that the lipid metabolic process of senescent cells promotes both proliferation and malignant progression of tumor cells, and at the same time, the lipid metabolic reprogramming of the tumor microenvironment also promotes the senescence of nontumor cells. Senescence may have some promoting effect on its lipid metabolism reprogramming.

### 3.7 Abnormalities of amino acid metabolism in tumor cells

In addition to amino acids being used as substrates for protein synthesis, they also support cancer cell growth as metabolites and metabolic regulators. Amino acid metabolism has an extremely wide‐ranging impact in cancer cells, which, in order to meet their rapid proliferation, must create pools of amino acids as building blocks for protein biosynthesis. These amino acids provide bioenergy into the tricarboxylic acid (TCA) cycle by producing α‐keto acids to generate ATP, which is converted to lipids and nucleosides. Amino acids also act as nutritional signals or neurotransmitters to activate important pathways and play an important role in epigenetic modifications.^133^ In addition to this, these biomolecules maintain the intracellular redox state by producing the antioxidant glutathione, such as the nonenzymatic cellular antioxidant glutathione synthesized from glutamate, cysteine, and glycine.

Amino acids can be categorized into two groups: nonessential amino acids, such as glutamate, glutamine, serine, and glycine; and essential amino acids, such as arginine, leucine, and methionine, the most prominent of which have been studied on glutamine, serine, and glycine.^134^ Increased glutamine metabolism is a common abnormality of amino acid metabolism in cancer. Glutamine, as the most abundant free amino acid, is second only to glucose in terms of its nutrient importance in cancer, and participates in a number of pathways such as energy production, macromolecule synthesis, and signaling in cancer cells by supplying nitrogen and carbon. Glutamine is imported into cancer cells via a variety of transporters, including Na+‐dependent transporters, systemic ASCs that act as obligatory exchangers (alanine/serine/cysteine preference), and Na+‐coupled neutral amino acid transporters^135^ belonging to the SLC38 superfamily, and inhibition of transporters has been shown to reduce the growth of a variety of cancers. Serine and glycine are interconnected during biosynthesis and are used together as basic precursors for synthesizing proteins, nucleic acids, and lipids that are essential for cancer proliferation to replenish energy. For example, Maddocks et al.^136^ found that limiting serine and glycine intake inhibited tumor growth and prolonged survival time in hormonal mice, and Camilla Tombari et al.^137^ found that the missense mutant p53 oncoprotein stimulated serine/glycine synthesis and essential amino acid uptake to promote breast cancer growth.

### 3.8 Abnormal amino acid metabolism in senescent cells

The experimental results of a study to measure the concentrations of metabolites in plasma of young and old people revealed an increased tendency to convert glutamate to glutamine during aging, and the systemic production rates of glutamine and tau‐methylhistidine in old people had WBP values 40% higher than normal, and that of arginine was 26% lower than normal. Whereas arginine clearance was reduced in the elderly, glutamine and leucine clearance was elevated.^138^ This confirms the alteration of amino acid metabolism in aging organisms and cells. In addition, the pathways by which amino acid metabolism is reprogrammed in aging cells are diverse and can be broadly categorized into direct and indirect approaches. Directly, amino acid metabolism is associated with aging through interrelated amino acid biosynthetic pathways, with amino acid metabolizing enzymes being the most representative class of age‐altered proteins. It has been found that glutamate synthase Glt1 folds and then polymerizes during aging to program supramolecular self‐assembly, further disrupting cellular amino acid homeostasis. This process is reversible, and inhibition of Glt1 polymerization restores amino acid levels in senescent cells, thereby extending lifespan. The senescent uterine microbiota may regulate cell cycle and apoptosis through microbial metabolites that are primarily involved in amino acid metabolism and biosynthesis of various secondary metabolites.^139^ Indirectly, key intermediates of amino acid metabolism are also strongly associated with senescence,^140^ such as s‐adenosyl‐l‐methionine (SAM), a key intermediate of sulfur amino acid (SAA) metabolism that is able to ultimately alleviate bone marrow mesenchymal stem cells (MSCs) senescence by activating PI3K/AKT signaling and increasing phosphorylation of FOXO3a, leading to a decrease in FOXO3a activity. In conclusion, the effect of senescence on amino acid metabolism is crucial, while further suggesting that senescence‐induced metabolic disorders may be potential therapeutic targets.

### 3.9 Abnormalities of amino acid metabolism in tumor‐promoting senescent cells

We have elaborated the abnormalities of amino acid metabolism in tumor cells and senescent cells, respectively, and further we wondered whether there is some connection between senescence, tumor, and amino acid metabolism. Based on the existing findings, we inferred that abnormalities in amino acid metabolism are triggered during aging, which in turn promotes tumor growth. For example, Xu et al.^141^ found that in HNF4α‐positive epithelial hepatocellular carcinoma cell lines, knockdown of HNF4α or SAA enzyme impaired hepatic SAA metabolism, increased resistance to methionine restriction or sorafenib, promoted epithelial–mesenchymal transition, and induced cell migration. Meanwhile, animal experiments have shown that aberrant methionine restriction metabolism can promote cancer growth by reducing lifespan through multiple signaling pathways. Aging, on the other hand, is a complex process of progressive physiological changes that affect biological functions over time and reduce quality of life,^141^ further affecting every metabolic pathway in adaptive and innate immune cells, ultimately leading to changes in immune cell function and alterations in the microenvironment. In turn, structural and functional changes in the components of the immune system lead to increased susceptibility to infections, autoimmune diseases, and cancer in older adults.^142^ Overall, pathways such as amino acid metabolism in immune cells regulate adaptive and innate immune cell differentiation and response,^143^ and these pathways are altered during aging, which in turn increases susceptibility to many diseases and thus promotes tumorigenesis. Currently, the research on aging, tumor and amino acids is not in‐depth, but by combining the above studies, we initially infer that abnormalities in amino acid metabolism are triggered during aging, which further promotes tumorigenesis.

Signaling pathways related to amino acid metabolism mainly include PI3K–mTOR pathway and AMPK pathway, which play important roles in regulating cell growth, metabolism, and stress response, and are closely related to aging and tumor. PI3K is a key coordinator of intracellular signaling in response to extracellular stimuli, and overactivation of the PI3K signaling cascade is one of the most common events in human cancer. Rapamycin complex 1 (mTORC1)^144^ is an intracellular protein complex that controls biosynthesis including proteins, lipids, and nucleic acids by integrating signals from growth factors and nutrients thereby. mTORC1 activity increases in response to the presence of amino acids and promotes cellular growth and protein synthesis while inhibiting autophagy. Another complex, mTORC2, is insensitive to rapamycin and, when active, regulates the actin cytoskeleton that controls cell shape and movement and can inhibit apoptosis. The energy sensor AMPK is considered a master regulator involved in the regulation of cellular metabolism in the presence of nutritional fluctuations, and the AMPK pathway is activated when intracellular ATP levels are low or AMP levels are high. Although AMPK is primarily associated with energy metabolism, changes in amino acid levels also affect its activity, thereby influencing cellular energy homeostasis and survival.^145^

## 4 MITOCHONDRIAL DYSFUNCTION AND TUMORIGENESIS

In recent years, as people continue to deepen the study of aging, researchers have found that mitochondrial dysfunction will cause cells to accelerate and consume a lot of energy. In the long run, this high metabolism of the cell will be faster than ordinary cell aging. This confirms what people have long suspected: mitochondrial function is closely related to aging. Mitochondria serve as pivotal actors in the theater of cellular processes, with their primary role being the powerhouse that supplies energy via oxidative phosphorylation and engages in the intricate dance of cellular metabolism. The primary metabolic processes taking place within mitochondria include the citric acid cycle, the breakdown of fatty acids through β‐oxidation, and the generation of ATP via oxidative phosphorylation. Due to the property of tumor cells to preferentially undergo aerobic glycolysis rather than oxidative phosphorylation for energy, researchers have speculated whether mitochondrial dysfunction is the structural basis for aerobic glycolysis in cancer cells. A substantial and growing body of scientific literature has provided compelling evidence supporting the association between mitochondrial dysfunction and the intricate process of tumorigenesis. Mitochondrial dysfunction refers to an impairment in the regular functioning of the mitochondria, which are essential cell organelles responsible for generating ATP and maintaining cellular energy balance. Numerous studies have indicated that dysregulation of mitochondrial function can lead to assorted pathological consequences, including the promotion of tumorigenesis. This disruption contributes to a host of cellular issues, which manifest in the form of altered metabolism, increased production of ROS, and initiation of apoptosis. It is within this context of mitochondrial perturbation that the correlation with tumorigenesis becomes particularly pronounced.^146^, ^147^, ^148^ Furthermore, mitochondrial dysfunction is widely acknowledged as a key feature of the aging process, and it stems from a multitude of contributing factors (Figure 5B): anomalies in the enzymes of the TCA cycle,^149^ the onslaught of oxidative stress,^150^ alterations in the mitochondrial DNA (mtDNA),^151^ impairments within the electron transport chain,^152^ and irregularities in gene expression. Below we will describe the causes of mitochondrial dysfunction and the impact of these causes on tumorigenesis.

### 4.1 Abnormalities of glucose metabolism occurring in mitochondria

#### 4.1.1 TCA cycle enzyme defects

A multitude of research efforts has revealed that alterations in the enzymes integral to the TCA cycle, such as succinate dehydrogenase,^153^, ^154^ can spur the emergence of diverse types of cancer, including hereditary pheochromocytoma,^155^ colorectal cancer,^156^ kidney cancer,^157^ gastrointestinal mesenchymal tumor,^158^ pituitary tumors,^159^ and ovarian cancer.^160^ Alterations in these enzymes trigger an accumulation of succinate and fumarate within the mitochondria. This build‐up impedes the actions of HIF‐1α prolyl hydroxylases,^161^, ^162^ culminating in the reinforced presence of HIF‐1α.^155^, ^163^ Consequently, this process augments the activation of genes crucial for mixed cellular functions, including cell proliferation, metabolic processes, angiogenesis, and the metastatic potential of cells, thereby setting the stage for the initiation and progression of cancer. Additionally, defects in TCA cycle enzymes^164^ serve as a catalyst for the production of ROS^149^, ^165^ and a concomitant escalation in mitochondrial oxidative stress, contributing further to the cellular environment conducive to oncogenesis. This intricate interplay underscores the intricate mechanisms by which dysregulation at the molecular level can pave the way for malignant transformation in biological systems.^166^ Elevated ROS levels within cancer cells^166^, ^167^, ^168^ are linked to genetic instability,^169^, ^170^ which, in turn, fuels the proliferation of these malignant cells.

#### 4.1.2 Oxidative stress

Oxidative stress is a complex physiological process marked by an excessive generation of highly reactive molecular species such as ROS^149^ and reactive nitrogen species.^171^, ^172^ This state of oxidative imbalance^173^ occurs when there is a considerable disparity^149^ between the production of these reactive molecules and the capacity of the body's antioxidant defense systems to counteract them. This imbalance can lead to cell and tissue damage and is implicated in multifaceted pathological conditions such as neurodegenerative diseases, cancer, and cardiovascular disorders. As a consequence, this imbalance can lead to the excessive oxidation or unchecked accumulation of potent free radicals^171^ within biological systems. The persistent presence of these radicals can inflict substantial damage upon cellular structures and biomolecules. Mitochondria are the main target of oxidative stress, and prolonged exposure to oxidative stress^174^ leads to impaired mitochondrial function,^171^, ^175^ which in turn triggers cellular senescence. Additionally mitochondria, when subjected to oxidative stress, release molecules that cause inflammation. These escalated concentrations of ROS^176^ within the neoplastic cells have a substantial immunomodulatory effect—affecting the tumor microenvironment^150^, ^177^ in a way that impairs T‐cell‐mediated immune responses. This impairment effectively creates an immune‐evasive landscape, thereby contributing to the further advancement of the malignant process.^178^, ^179^ The capacity of cancerous cells to circumvent the body's natural immunosurveillance mechanisms by leveraging heightened ROS levels represents a considerable hurdle in oncology, further complicating the intricate interplay between host defenses and neoplastic progression.

#### 4.1.3 mtDNA mutations

Several studies have indicated a potential correlation between mtDNA mutation and specific forms of tumor growth.^180^, ^181^ Interestingly, these same mutations can occasionally be identified within both healthy and cancerous tissues.^182^ This raises the question of whether every mtDNA mutation truly contributes to the onset of cancer. The possibility of a definitive connection between mtDNA mutation and the development of cancer necessitates comprehensive and meticulous research in the future. Contemporary research on mtDNA aberrations in cancerous cells primarily underscores the correlation between mtDNA mutations and elevated levels of free radicals^151^ and increased levels of ROS in tumor cells, stimulating tumor cells to develop proliferative and invasive properties; mtDNA mutations can also trigger mitochondrial respiratory chain dysfunction, affecting electron transfer, oxygen consumption, and ATP generation.^182^ Mutations in mtDNA can also trigger mitochondrial respiratory chain dysfunction, affecting electron transfer, oxygen consumption, and ATP generation.

#### 4.1.4 Electronic transfer chain defects

The mitochondrial electron transport chain, a crucial component of cellular respiration, is constituted through the intricate assembly of protein subunits. These subunits, which play a crucial role in cellular function, are the result of a unique collaboration between the genetic information contained within the cell nucleus and the mitochondria. The nuclear DNA encodes the majority of the proteins that make up these subunits,^183^ while the mtDNA^184^ also contributes to the synthesis of a specific set of subunits essential for the proper functioning of the mitochondria.^185^ This collaborative process exemplifies the intricate relationship between nuclear DNA and mtDNA in orchestrating the complex molecular machinery of the cell. Such synergy between the two types of DNA highlights the interdependence and coordinated effort required for the production of functional mitochondrial subunits, underscoring the interconnected nature of genetic information within the cell. The process of assembling these subunits into a functional electron transport chain is not only complex but also necessitates a host of diverse assembly cofactors.^183^ Mutations within mtDNA are known to disrupt the mitochondrial electron transport chain,^152^ leading to faults in how cells manage their energy currency,^186^ ATP. Research has consistently found that these mutations can impair diverse components of the electron transport chain, which in turn ramps up the production of mitochondrial reactive oxygen^187^ species. This surge in ROS is coupled with a rise in lactate production,^185^, ^188^, ^189^ fostering a climate that advances the malignant progression of multiple cancers like pancreatic cancer,^185^ osteosarcoma,^190^ breast cancer,^191^ and colorectal cancer.^192^

#### 4.1.5 Abnormal expression of oncogenes and oncogenes

Alterations within KRAS, a pivotal constituent of the RAS family of small guanosine triphosphatases, are exceedingly prevalent in a myriad of human malignancies. Notably, mutations in specific alleles of the KRAS gene^193^ are detected in an overwhelming majority, exceeding 95%, of cases of pancreatic ductal adenocarcinomas, underscoring the significance of this genetic aberration in the pathogenesis of this lethal disease.^194^, ^195^ The overexpression of a mutant form of KRAS critically disrupts the functionality of mitochondria, precipitating an escalation in mitochondrial oxidative stress,^196^, ^197^, ^198^ which in turn plays a pivotal role in the facilitation of tumorigenesis. Furthermore, the expression of an oncogenic variant of KRAS is closely associated with a marked increase in glycolytic activity^199^, ^200^ within the cells,^201^ thereby substantiating a metabolic reprogramming that fosters an environment conducive to tumor cell proliferation and survival.

Complementary to KRAS‐induced metabolic disturbances, the oncogene c‐Myc represents another formidable architect of the tumoral metabolic landscape, chiefly by augmenting aerobic glycolysis.^202^ This is achieved through the enhanced transcription of a cadre of critical genes involved in the glycolytic pathway.^202^, ^203^, ^204^ Such metabolic reprogramming endorsed by c‐Myc not only caters to the energetic^203^ and biosynthetic requirements of rapidly dividing tumor cells but also contributes to the reshaping of the tumor microenvironment.

Concurrently, c‐Myc is implicated in the promotion of oxidative stress within tumor cells,^205^, ^206^, ^207^ a condition that, while deleterious to normal cellular function, paradoxically may contribute to the malignant phenotype by inflicting DNA damage and fostering genomic instability. Furthermore, c‐Myc is known to upregulate the NF‐κB signaling pathway.^205^ Collectively, the dysregulation of KRAS and c‐Myc exemplifies the intricate molecular interplay that underlies the emergence and progression of cancer, spotlighting potential therapeutic targets for intervention in this complex and devastating disease.

### 4.2 Abnormal peroxisome and mitochondrial function in lipid metabolism inhibits cellular senescence

In normal cellular lipid metabolism, most of the β‐oxidation of fatty acids takes place in the mitochondria, while the remaining 25–50% of fatty acids are oxidized in the peroxisome. In 2016, mitochondrial dysfunction‐associated senescence (MiDAS), defined by Wiley et al.,^208^ was experimentally shown to induce cellular senescence with a unique secretory pattern. Senescence and mitochondrial dysfunction interact with each other in a causal manner.^209^ TRPC3 protein remodels Ca^2+^ release leading to mitochondrial dysfunction, which promotes protumorigenic effects in senescent cells.^210^ Based on the above studies, we can find that mitochondrial dysfunction interacts with senescent cells, and at the same time, mitochondrial dysfunction further enhances its tumor‐promoting effects through its action on senescent cells.

Peroxisomes are conserved organelles in eukaryotes, which play an important role in lipid metabolism as well as the degradation of ROS in cells. Peroxisome dysfunction will lead to the development of a wide range of diseases including neurodegenerative diseases, age‐related metabolic disorders and others. The evidence that peroxisome activity declines with age while leading to age‐related diseases^211^ seems to suggest that peroxisome dysfunction can similarly promote cellular senescence. As far as the current study is concerned, the role of peroxisome dysfunction for tumors is unclear. Some reports suggest that whether peroxisomes promote or inhibit tumors depends on the specific tumor type.

### 4.3 Abnormalities of amino acid metabolism occurring in mitochondria

Amino acids are intricately linked to mitochondria, influencing their bioenergetic, synthetic, and homeostatic functions. Most amino acid metabolic pathways span both mitochondrial and cytoplasmic locations, and their pathways are localized in a tissue‐specific manner,^139^ for example, cobalamin metabolism undergoes a transamination pathway in the cytoplasm and a transdeamination pathway in mitochondria; serine metabolism participates in a phosphorylation pathway in the cytoplasm, whereas the nonphosphorylation pathway occurs in mitochondria; and glycine metabolism is formed through two pathways, the first of which is formed from serine via the hydroxymethyltransferase, which occurs in both cytoplasmic and mitochondrial locations. The second pathway is catalyzed by the formation of glycine synthase, which occurs in the mitochondria. Abnormalities in amino acid metabolism are closely associated with mitochondrial dysfunction and may affect mitochondrial translation, the TCA cycle, and mitochondrial iron–sulfur cluster proteins and thus lead to a variety of human diseases, for example, abnormalities in arginine metabolism can lead to guanidinium butyl ester methyltransferase deficiency, argininemia, ornithine transcarbamoylase deficiency, and ornithinemia with rotational atrophy of the choroid and retina, among other disorders.^212^

Amino acids require specific transporters to facilitate their import, export, and exchange within the mitochondrial membrane, and thus mitochondrial transporter proteins may play a key role in promoting amino acid metabolic activity. Mitochondrial amino acid carriers include adenine nucleotides, amino acids, acylcarnitines, and small organic acids, of which SLC25 is the largest component of mitochondrial transporter proteins, with 53 members of the family. New advances have recently been made in the identification of mitochondrial amino acid carriers in the SLC25 family, such as the SLC1A5^213^ variant, which is a mitochondrial glutamine transporter used for metabolic reprogramming in cancer; SLC25A22^214^ is a key mitochondrial transporter protein that counteracts iron death by producing glutathione and monounsaturated fatty acids. Cytoplasmic and mitochondrial amino acid exchanges facilitated by mitochondrial transporters are critical for redox shuttle activity^215^ but a number of amino acid transporter proteins are unknown, including asparagine, tryptophan, alanine, methionine, phenylalanine, tyrosine, cysteine, and proline. Recent new techniques for more precise quantification of mitochondrial metabolism, transport and metabolite synthesis can shed light on whether mitochondrial transport occurs and which transporters are involved.^213^, ^216^

In summary, mitochondrial dysfunction plays a key role in tumorigenesis, and further insight into the specific mechanisms by which mitochondrial dysfunction promotes tumor progression will help us to develop new therapeutic approaches.

## 5 CELLULAR SENESCENCE AND APOPTOSIS CAUSED BY MITOCHONDRIAL DYSFUNCTION

It is well known that as cells age, the ability of cell proliferation and differentiation, and physiological functions gradually decline, which will eventually lead to cell death. Depending on whether it is controllable or not, cell death can be divided into two major categories, that is, programmed death and necrosis. Among them, programmed cell death includes apoptosis, pyroptosis, autophagy, necroptosis, iron death, copper death, and other forms. The most studied type of cell death is apoptosis. Cellular senescence and apoptosis are two different biological processes. Cellular senescence is a degenerative process, mainly related to pathological conditions associated with aging, and is usually triggered by a stress response due to damaging stimuli or abnormal proliferation. Apoptosis, on the other hand, is an active and orderly cellular suicide process, usually caused by various stimuli inside and outside the cell, such as DNA damage, oxidative stress, and mitochondrial dysfunction. These stimuli will activate the apoptotic signaling pathway in the cell, which will eventually lead to autonomous cell death.^217^

In the previous section, we summarized the senescence‐related markers, in which the relationship between the SASP and apoptosis drew our attention. Most of the SASP secreted by senescent cells contain proapoptotic factors, such as interferons like IFN‐α and IFN‐β, proinflammatory cytokines like TNF‐α, IL‐1β, and IL‐6, as well as chemokines such as GM‐CSF, CCL2, CCL3, CCL4, and so on. These SASP components can be activated by activation of immune cells (e.g., NK cells, macrophages, etc.), inducing cell membrane. These SASP components can induce cell membrane damage by activating immune cells (e.g. NK cells, macrophages, etc.) and ultimately lead to apoptosis of defective senescent cells.^218^, ^219^ In addition, some components of SASP, such as exosomes and ROS, can directly damage the cell membrane and lead to apoptosis. In other senescent cells, SASP seems to contain growth VEGF‐A and PDGF‐AA and other regenerative factors,^219^, ^220^ which may reduce apoptosis, tissue damage, and fibrosis compared with proapoptotic, proinflammatory senescent cells. In conclusion, there is a complex interrelationship between SASP and apoptosis. SASP can induce apoptosis through activation of immune cells or direct damage to cell membranes, but may also inhibit apoptosis in some cases, thereby promoting the propagation of the senescent phenotype.

Current research on the relationship between senescence and apoptosis focuses on the apoptosis resistance characteristics of senescence. During cellular senescence, some antiapoptotic signaling pathways, so‐called “senescent cell antiapoptotic pathways (SCAPs),” are activated. During senescence, cellular resistance to apoptosis increases, resulting in the survival of functionally defective aging cells. This “age‐related apoptosis resistance” may accelerate the aging process.^221^ Among these SCAPs, we first noticed the PI3K–AKT–mTOR signaling pathway, which is also found in the aerobic glycolytic signaling pathway in tumour cells and in the glycolytic signaling pathway in senescent cells. Studies have shown that activation of the mTOR pathway promotes the expression of the inflammatory factor IL‐1A, thereby enhancing SASP.^222^ This enhancement of SASP can improve the antiapoptotic ability of senescent cells.^82^ On the other hand, long‐term activation of the mTOR pathway leads to the loss of mTORC2, which causes insulin resistance,^223^ which in turn decreases the antiapoptotic capacity of cells.^82^ Besides, changes in p53 activity also affect apoptosis and senescence. On the one hand, p53 induces the expression of proapoptotic Bcl‐2 family proteins and inhibits the expression of antiapoptotic Bcl‐2 and Bcl‐xL genes in a transcription‐dependent manner, thereby activating apoptosis^224^, ^225^, ^226^; meanwhile, p53 can also interact directly with Bcl‐2 family proteins in a nontranscription‐dependent manner, regulating the permeability of the outer mitochondrial membrane,^222^ which in turn reduces the antiapoptotic capacity of cells.^223^ At the same time, p53 can also interact directly with Bcl‐2 family proteins in a nontranscription‐dependent manner to regulate the permeability of the outer mitochondrial membrane, promote the release of apoptotic factors, such as cytochrome *c*, and induce cell apoptosis.^221^ Apoptosis induced by activation of p53 can accelerate the aging process. On the other hand, with the increase of age, p53 activity decreases, and p53‐dependent apoptotic response will be weakened, leading to an increase in cellular resistance to injury, thus promoting aging. p53 activity decreases will also lead to alterations in cellular metabolism, increase glycolytic,^227^ and NF‐κB^228^ pathway activity, which will enhance the cellular resistance to apoptosis and slow down aging.

So, senescence and apoptosis present a complex relationship. SASPs can induce or reduce apoptosis. Apoptosis plays an important role in organismal development and maintenance of cellular homeostasis, while changes in cellular resistance to apoptosis are an important mechanism leading to senescence.

## 6 THERAPEUTIC MEASURES THAT HAVE ENTERED THE CLINICAL PHASE

Presently, the primary approaches to treating tumors encompass surgical intervention, comprehensive chemotherapy, radiation therapy, therapy targeting specific molecules, and immunotherapy. In contrast to conventional chemotherapy treatments, targeted therapies offer a more accurate approach, minimizing harm to healthy cells while enhancing the efficacy of the treatment. ClinicalTrials.gov, an extensive database that has risen to global preeminence amongst clinical trial registries, was a collaborative venture created through the concerted efforts of the US National Library of Medicine and the US Food and Drug Administration. Conceived in 1997, this groundbreaking resource was officially unveiled in February 2002. Since its inception, it has been disproportionately influential in shaping the landscape of clinical research, and is, to date, the most prolifically utilized registry of its kind. In this database, we have comprehensively distilled the cancer‐related clinical trials that included participants aged 65 years and above. Our aim was to pinpoint studies that simultaneously addressed the issues of “aging” and “tumor.” There are only eight signaling pathways targeted by such studies, namely, the PI3K/mTOR, JAK–STAT, GH/IGF‐1, NF‐κB, Hippo, Wnt/β‐catenin, Notch, and AMPK signaling pathways; detailed data on completed and ongoing clinical trials are summarized in Table 1.

**TABLE 1:** Targeted drugs that inhibit aging and tumor signaling pathways.

Targeting signaling pathways	Drug use	Tumor type	Clinical phase	Clinical serial number
PI3K/mTOR	MSC1936369B (pimasertib)	Lymphoma	Phase 1	NCT01390818
	BEZ235	Malignant solid tumors	Phase 1	NCT01343498
	AZD2014, anastrozole	Endometrial cancer, estrogen receptor positive breast cancer	Phase 1	NCT02730923
	Rapamon, apatinib	Progressive solid tumors	Phase 1	NCT00337376
	RAD001, RG1507	Progressive solid tumors	Phase 1	NCT00985374
	Capecitabine, RAD001	Lymphoma	Phase 1	NCT00473005
	Everolimus (RAD001)	Prostate cancer patients with detectable PSA after prostatectomy	Phase 1	NCT01548807
	Everolimus (loanword)	Locally advanced cervical cancer	Phase 1	NCT01217177
	Hydroxychloroquine, RAD001	Metastatic clear cell renal cell carcinoma	Phase 1	NCT01510119
	AstraZeneca, olaparib	BRCA1 gene mutation carriers, BRCA2 gene mutation carriers, endometrial adenocarcinoma	Phase 1/2	NCT02208375
	AZD2014	Glioblastoma multiforme	Phase 1	NCT02619864
	MLN0128	Metastatic anticancer prostate cancer	Phase 2	NCT02091531
	Exemestane, PF‐04691502	lymphoma	Phase 2	NCT01658176
	Everolimus (loanword)	Kaposi's sarcoma	Phase 2	NCT01412515
	Sirolimus albumin‐bound nanoparticles	Neuroendocrine tumors, pancreatic neuroendocrine tumors	Phase 2	NCT05997056
	Smetinib, sirolimus	Malignant peripheral nerve sheath tumor	Phase 2	NCT03433183
	Sirolimus (loanword)	Progressive solid tumors	Phase 2	NCT00877773
	Isomethepromazine, everolimus	Her2 negative metastatic breast cancer	Phase 2	NCT01520103
	Anastrozole, AZD2014	Endometrial cancer, estrogen receptor positive breast cancer	Phase 2	NCT02730923
	pf‐05212384, pf‐05212384, pf‐05212384	Endometrial cancer	Phase 2	NCT01420081
	Sirolimus (loanword)	Ovaries	Phase 2	NCT00926107
	Hydroxychloroquine (antimalarial drug)	Metastatic clear cell renal cell carcinoma	Phase 2	NCT01510119
JAK–STAT	INCB052793, gemcitabine, albumin paclitaxel	Malignant solid tumors	Phase 1/2	NCT02265510
	Pactinib	Colorectal cancer	Phase 2	NCT02277093
GH/IGF‐1	Growth hormone	Chronic granulocytic leukemia	Phase 4	NCT01901666
NF‐κB	Epidiferphane	Breast cancer	Phase 1	NCT03611985
	Taxane ASTX660 Pembrolizumab	Terminal cancer Cervical cancer Triple‐negative breast cancer	Phase 1	NCT05082259
	Curcumin	Multiple myeloma	Phase 2	NCT01269203
	Vorinostat bortezomib Placebo to vorinostat	Multiple myeloma	Phase 3	NCT00773747
	Valproic acid	Lymphomas	Phase 4	NCT00854581
STAT3	DCR–STAT3	Solid tumors	Phase 1	NCT06098651
	STAT3 inhibitor WP1066	Metastatic malignant tumor of the brain Metastatic melanoma Recurrent brain tumors	Phase 1	NCT01904123
	TTI‐101	Breast cancer Head and neck squamous cell carcinoma Non‐small cell lung cancer	Phase 1	NCT03195699
	AZD9150	Ovarian cancer	Phase 2	NCT02417753
	Nivolumab 10 MG/ML BNC 105 BBI608	Metastatic colorectal cancer	Phase 2	NCT03647839
Hippo	IK‐930 Osimertinib	Solid tumors Malignant pleural mesothelioma (MPM)	Phase 1	NCT05228015
	Atorvastatin	Breast tumor	Phase 2	NCT02416427
Wnt/β‐catenin	Artesunate 200 mg	Colorectal cancer	Phase 2	NCT02633098
	Sinecatechins 10%	Basal cell carcinoma	Phase 3	NCT02029352
Notch	Gamma‐secretase inhibitor RO4929097 Ketoconazole Rifampin	Solid tumors	Phase 1	NCT01218620
	AL101	Endometrioid plasmacytoid carcinoma Recurrent endometrial carcinoma Recurrent renal cell carcinoma	Phase 1	NCT01198184
	Gamma‐secretase/Notch signaling pathway inhibitor RO4929097	Estrogen receptor negative breast cancer HER2‐negative breast cancer	Phase 2	NCT01151449
	Gamma‐secretase/Notch signaling pathway inhibitor RO4929097	Male breast cancer Adult alveolar soft part sarcoma Adult angiosarcoma Adult connective tissue proliferative small round cell tumor	Phase 2	NCT01120275
AMPK	6‐Mercaptopurine	Malignant glioma	Phase 1	NCT06279767
	Placebo	Hepatocellular carcinoma	Phase 1	NCT02261844
	Metformin rapamycin	Breast tumor Lung tumor Liver cancer	Phase 1	NCT02145559
	Sunitinib Isoquercetin placebo	Renal cell carcinoma Kidney cancer	Phase 1	NCT02446795
	Aspirin	Melanoma (skin)	Phase 1	NCT04062032
	Metformin	Breast cancer	Phase 1	NCT01266486
	Metformin	Endometrial cancer	Phase 2	NCT02042495
	Metformin placebo	Chemotherapy‐induced peripheral neuropathy	Phase 2	NCT04780854
	Lapatinib metformin	Metastatic breast cancer	Phase 2	NCT01477060
	Metformin placebo	Cushing's disease	Phase 2	NCT01319994
	Metformin hydrochloride placebo	Prostate adenocarcinoma	Phase 2	NCT01433913
	Metformin	Non‐small cell lung cancer	Phase 2	NCT02019979
	Gemcitabine erlotinib Metformin placebo	Locally advanced pancreatic cancer Metastatic pancreatic cancer	Phase 2	NCT01210911
	Letrozole abemaciclib, LY3023414 Metformin zotarfen	Endometrial cancer	Phase 2	NCT03675893
	Everolimus plus octreotide LAR plus metformin	Highly differentiated pancreatic endocrine tumors	Phase 2	NCT02294006
	Metformin Rituximab Cyclophosphamide Doxorubicin Vincristine Prednisone Polyfilgrastim	Diffuse large b‐cell lymphoma	Phase 2	NCT02531308

Data in table from ClinicalTrials.gov.

From the table, we can see that there are more clinical trials on aging and tumor‐related signaling pathways, and the types of tumors treated are very rich, the drugs used are mainly everolimus, sirolimus, anastrozole, and so on, and there are more experimental protocols of combination drugs in recent years, which suggests that the combination of drugs is the focus area of tumor therapy and antiaging research in the future.

## 7 NANOTARGETING STRATEGIES WITH THERAPEUTIC POTENTIAL

**FIGURE 6:**
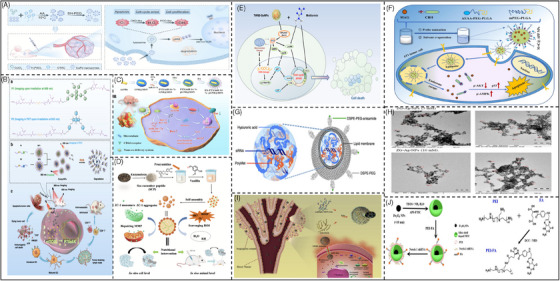
Regulation of signaling pathways associated with organismal aging and tumorigenesis by nanomaterials. (A) Two‐dimensional copper–palladium nanoenzymes modulate aberrant activation of the PI3K/AKT/mTOR pathway p85β to reverse tamoxifen resistance in breast cancer.^230^ Copyright 2023, Elsevier. CuPd nanoenzymes in 2D form are effective at overcoming tamoxifen resistance by producing reactive oxygen species, which degrade the p85β protein subunit of PI3K and suppress the PI3K/AKT/mTOR signaling pathway. (B) Therapeutic imaging using the mTOR signaling pathway for multimodal photodynamic therapy and immunotherapy.^231^ Copyright 2023, Springer Nature. The highly efficient chromophore M1 and the photosensitizer M2 were structurally modified by adding hydroxyl functional groups. This led to the self‐assembly of polymers into nanoparticles NP1 and NP2.^231^ The therapeutic nanoparticle formulation Comp‐NPs consists of a combination of NP1 and NP2. (C) Novel targeted codelivery nanosystems enhance ovarian cancer therapy through multidrug resistance reversal and mTOR‐mediated signaling pathways.^232^ Copyright 2021, Springer Nature. A new delivery system, called HA–PTX/let‐7a‐GNR@MSN,^232^ simultaneously delivers the hydrophobic antitumor drug paclitaxel and the gene drug miR‐let‐7a, targeting mTOR‐mediated signaling pathways, using novel hyaluronic acid (HA)‐targeted modified mesoporous silica‐coated gold nanorods. (D) Extension of lifespan of loaded mice by size‐controlled food‐grade nanoparticles based on sea cucumber peptides with good antioxidant capacity.^233^ Copyright 2023, Elsevier. Vanillin furnished the necessary aldehyde for producing SCP and PC nanoparticles^233^ through the Mannich reaction: vanillin. SCP‐based nanoparticles (SCP‐NPs) derived from sea cucumber polypeptide were fabricated by covalently assembling proanthocyanidins (PCs) with vanillin in a one‐pot Mannich condensation process. (E) Functionalized selenium nanoparticles synergizes with metformin to treat breast cancer cells through regulation of selenoproteins. Front Bioeng Biotechnol.^234^ Copyright 2021, CC‐BY 4.0. (F) Magnolol‐loaded cholesteryl biguanide conjugate hydrochloride nanoparticles for triple‐negative breast cancer therapy.^235^ Copyright 2022, Elsevier. (G) PolyMetformin combines carrier and anticancer activities for in vivo siRNA delivery.^236^ Copyright 2016, Springer Nature. (H) Synthesis of Ziziphus spina‐christi (Jujube) root methanol extract‐loaded functionalized silver nanoparticle (ZS‐Ag‐NPs); physiochemical characterization and effect of ZS‐Ag‐NPs on adipocyte maturation, adipokine and vascular smooth muscle cell interaction.^237^ Copyright 2021, CC‐BY 4.0. (I) HA‐chitosan nanoparticles for codelivery of MiR‐34a and doxorubicin in therapy against triple negative breast cancer.^238^ Copyright 2014, Elsevier. (J) Multifunctional core/shell nanoparticles cross‐linked polyetherimide‐folic acid as efficient Notch‐1 siRNA carrier for targeted killing of breast cancer.^239^ Copyright 2014, Springer Nature.

The relationship between aging and tumors as mutual targets has been studied not only by the above mentioned studies that are already or will soon be in the clinical stage, but also by those that are currently in the research and development stage. In recent years, nanotargeted materials have highlighted a greater potential for drug/gene delivery. Researchers have found that nanomaterials, due to their own ultra‐permeable and accumulating retention effects in lymphoid tissues.^229^ Nanomaterials technology holds great promise for improving treatment outcomes for patients with metastatic cancers, as rationally designed nanomaterials have the potential to elicit targeted tumor‐killing effects, halt the formation of premetastatic niches, and inhibit tumor recurrence through postsurgical immunotherapy. Therefore, in this section we summarize the nanomaterials targeting the signaling pathways that inhibit organismal aging and tumorigenesis, hoping to inspire researchers for future clinical studies on aging and tumors. Currently, among the signaling pathways related to aging and tumor, only the PI3K/mTOR, AMPK, and Notch signaling pathway has been studied with relevant targeted nanomaterials, which are summarized in Figure 6. Most of these are biocomposites, in which researchers coat metallic or nonmetallic nanomaterials with biocompatible proteins or amino acids to achieve therapeutic effects.

1. The team discovered that overexpression of the PI3K subunit p85β protein can trigger the PI3K/AKT/mTOR pathway,^60^ leading to tamoxifen resistance. Consequently, in Figure 6A, they developed a 2D‐CuPd nano‐enzymes to produce ROS, which in turn broke down the PI3K subunit p85β protein and suppressed the PI3K/AKT/mTOR signaling pathway^230^ to overcome tamoxifen drug resistance.
2. Photodynamic therapy is performed by injecting a photosensitizer locally or systemically into the patient and exposing the tumor site to irradiation after a certain incubation time. Following light activation, in Figure 6B, the photosensitizer can effectively produce ROS through photocatalysis, leading to oxidative injury in the adjacent tissues, offering a targeted treatment for local tumors.^231^ Based on this principle, the group structurally modified the highly efficient chromophore and photosensitizer and assembled the resulting polymer into composite nanoparticles; the nanoparticles can accumulate at the tumor site and be activated by light irradiation to inhibit the mTOR signaling pathway, thus inhibiting the malignant progression and recurrence of the tumor.
3. Chemotherapy resistance, particularly multidrug resistance (MDR),^232^ is a major obstacle in effectively treating ovarian cancer. The mTOR signaling pathway has been identified as a significant contributor to the development of MDR in ovarian cancer. To combat this issue, in Figure 6C, a groundbreaking hyaluronic acid (HA)‐targeted modified mesoporous silica‐coated gold nanorods co‐delivery system^232^ has been devised. This innovative system is designed to transport the antitumor drug paclitaxel (PTX) and the gene drug miR‐let‐7a to selectively target the mTOR signaling pathway. By doing so, it can downregulate the expression of STAT3, ultimately impeding tumor proliferation. Additionally, miR‐let‐7a can effectively reduce the expression of P‐glycoprotein, potentially reversing the multidrug resistance typically encountered in ovarian cancer.
4. Sea cucumber polypeptides (SCPs) are derived from the hydrolysis of sea cucumber proteins using enzymatic cleavage. These peptides are composed of a range of essential and nonessential amino acids, which play vital roles in maintaining human health. These diverse amino acids in SCPs contribute to their potential health benefits,^233^ encompassing immune modulation, antioxidant activity, and anti‐inflammatory properties. SCPs, as vital components in sea cucumbers, can block the PI3K/AKT/mTOR signaling pathway.^240^ Nevertheless, producing SCP‐based nanodelivery systems of customizable sizes typically involves complicated processes and multiple raw materials. In Figure 6D, the team fabricated SCP‐based nanoparticles (SCP‐NPs) by covalently linking proanthocyanidins with a one‐step Mannich condensation method, which effectively decreased intracellular ROS and eliminated free radicals, thus extending the survival of mice with tumors through the clear.
5. In Figure 6E, selenium nanoparticles Tween 80 (TW80)‐SeNPs in combination with metformin have a synergistic effect on MCF‐7 cells, and the mechanism of this synergistic effect involves the induction of DNA damage through the influence of selenoproteins on ROS production, which further affects the expression of AMPK and ultimately inhibits tumor growth.^234^
6. In Figure 6F, researchers added poly(ethylene glycol)–poly(lactic acid)‐co‐hydroxyacetic acid and aminoethylene ester–poly(ethylene glycol)–poly(lactic acid)‐co‐hydroxyacetic acid to form nanoparticles, which altered the levels of p53, p‐AKT, and p‐AMPK in tumor tissues and were able to inhibit tumor growth without significant toxicity. Overall, this nanoparticle system provides a new co‐administration strategy for the treatment of triple‐negative breast cancer.^235^
7. In Figure 6G, LPH‐PolyMet nanoparticles act similarly to metformin in inducing antitumor effects through activation of AMPK and inhibition of mTOR.^236^
8. In Figure 6H, researchers synthesized ZSE silver nanoparticles (ZS‐Ag‐NPs) using methanolic root extract of Ziziphus spina‐christi (L.) with mitochondrial thermogenicity, which in turn significantly increased the levels of CREB‐1 and AMPK proteins in adipocytes, which ultimately could be effective in reducing the progression of obesity and the pathogenesis of metabolic inflammation associated with aging. Pathogenesis of metabolic inflammation associated with aging.^237^
9. In Figure 6I, Deng et al. had used chitosan nanoparticles to target delivery of miR‐34a into triple negative breast cancer tumor cells and found that miR‐34a not only inhibited tumors, but also reduced the expression of NOTCH‐1.^238^
10. In Figure 6J, Yang et al.^239^ used iron oxide‐silica synthesized nanoparticles to precisely target shRNA to NOTCH‐1, again effectively inhibiting the tumor.

**FIGURE 7:**
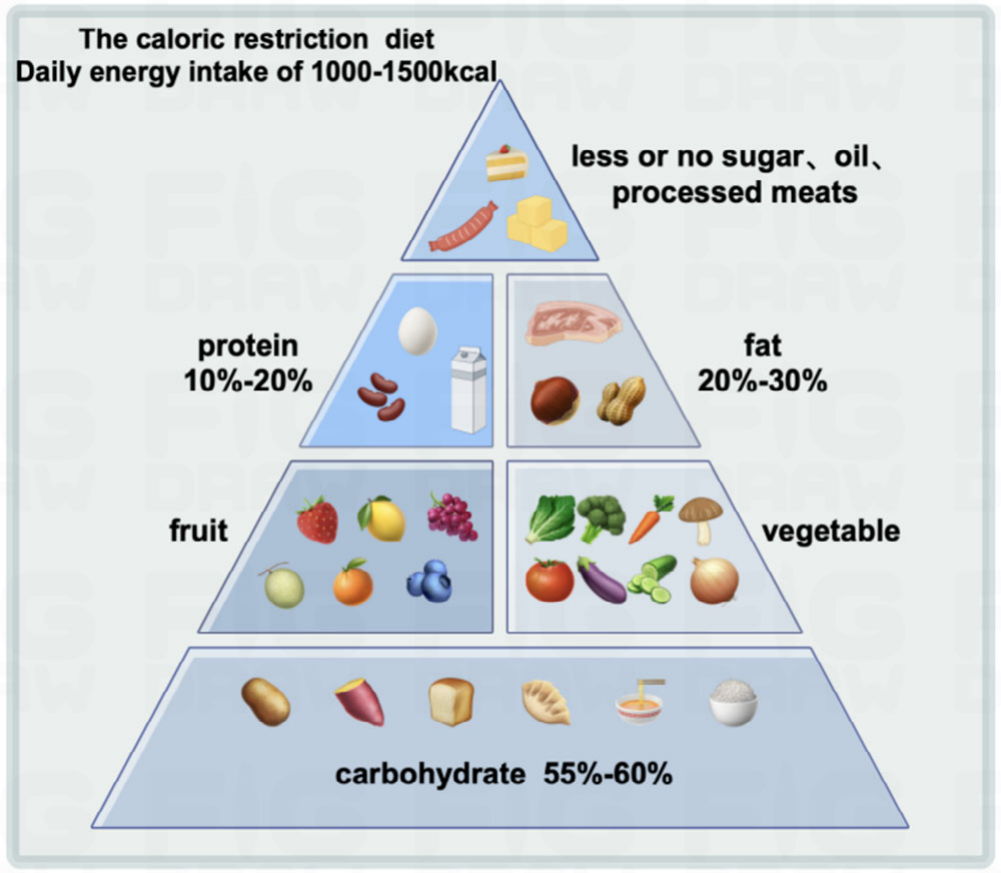
Dietary recommendations. For the antiaging and antitumor population, it is recommended to adopt the CR dietary pattern, with a daily intake of 1000–1500 kcal and a reasonable ratio of the three major nutrients; minimize the intake of sugar, oil, and processed meat; give priority to citrus and berry fruits and dark green vegetables; and eat more onions and tomatoes to replenish flavonoids and carotenoids. This figure is drawn by the Figdraw online platform, thanks to Figdraw (https://www.figdraw. com/static/index.html#/).

In conclusion, in terms of clinical‐stage therapeutic measures and developed nanotargeting strategies, existing studies have focused on the investigation of the PI3K/mTOR, AMPK, and Notch signaling pathway. It is hoped that more abundant research results will be available in the future to provide a theoretical basis for clinical treatment.

## 8 DIETARY ADVICE

Diet is an important part of human life, which, in addition to maintaining the normal vital signs of human beings, also reflects people's culture, health, and attitude toward life. As the social economy continues to progress and national living standards improve, there has been a discernible increase in the demand for food among the populace. Concomitantly, there is a heightened focus on the cultivation of dietary habits conducive to achieving and maintaining a state of optimal health. The burgeoning interest in healthy dietary practices can be attributed to a growing awareness of the profound impact that food consumption has on overall well‐being. This trend is underscored by a plethora of studies illuminating the intricate relationship between dietary choices and health outcomes, thereby driving individuals to be more vigilant and discerning in their culinary decisions. In this section, we will make some dietary suggestions for antiaging and antitumor.

### 8.1 Recommended dietary pattern

One of the most extensively studied and widely implemented approaches to antiaging nutrition is the caloric restriction (CR) diet.^241^, ^242^, ^243^ CR involves a significant reduction of 30–40% in total energy intake, and it is frequently achieved through practices such as intermittent fasting.^244^ A growing body of research has underscored the potential health benefits associated with CR, suggesting that restricting caloric intake may offer a promising avenue for combating age‐related decline and promoting longevity. Furthermore, CR has been associated with improved metabolic function,^245^ decreased inflammation,^246^ and enhanced cellular repair processes.^247^ Numerous studies have demonstrated that restricting food intake in animal models can significantly reduce their life span and tumor incidence.^248^, ^249^ The results of these studies have been shown to be significant in terms of lifespan and tumor incidence. Based on a large number of animal experiments, in 2022, *Science* published the first human clinical trial results of calorie‐restricted diet programs, which even proved that calorie‐restricted diets can prolong life. However, calorie‐restricted diet is not equal to excessive dieting, and since most people cannot adhere to it for a long time or have extreme diets, researchers have proposed a more scientific and reasonable dietary pattern, that is, daily energy supply of 1000–1500 kcal.^250^ And to ensure that the three major nutrients intake ratio is reasonable (carbohydrates accounted for 55–60% of the total daily energy, fat accounted for 20–30%, and protein accounted for 10–20%) (Figure 7).

### 8.2 Require special attention in terms of intake

In addition to fulfilling our daily dietary needs we can also target more phytochemicals. Phytochemicals are widely found in fruits, vegetables, cereals, nuts, cocoa/chocolate, fruit juices, tea, coffee, and so on, and are the biologically active constituents of plant‐based foods.^251^, ^252^ Phytochemicals include flavonoids, isoflavonoids, phenols and polyphenols, carotenoids, organosulfides, indoles, phytoandrogens, citrullinated compounds, lycopene, and phytosterols. Although phytochemicals are not essential nutrients, they have anticancer and antioxidant properties^253^, ^254^ and antioxidant^255^ and anti‐inflammatory^256^ functions. For example, supplementation of flavonoids and isoflavonoids can effectively eliminate free radicals in the cell.^251^ Daily consumption of beans, onions, and other flavonoids supplement is recommended as they are anti‐inflammatory, antiaging, and help in tumor inhibition. Second, epidemiological evidence shows that fruits and vegetables (especially dark green vegetables) can significantly prevent tumor development and slow down cellular aging.^257^ Eating more fruits and vegetables can supplement vitamin C,^256^ vitamin B2, folic acid, potassium,^259^ calcium, magnesium,^260^ iron, and dietary fiber. Consumption of tomato and spinach is recommended for prevention of tumors as their antiaging role is particularly prominent; large number of studies have proved that these two kinds of food in China are rich in carotenoids^261^ and can significantly reduce human suffering from lung cancer, stomach cancer,^262^ ovarian cancer, breast cancer, aging‐related cancers,^263^ and many other cancers, as well as aging‐induced macular degeneration and cataracts.^264^ Finally, meat is also indispensable in the daily diet,^265^ being rich in protein, fat, calcium,^266^ iron, and other nutrients. Previously, the International Agency for Research on Cancer (IARC), a specialized agency of the World Health Organization, recently conducted an extensive investigation into the health effects of consuming processed meat products and red meat. Their comprehensive report conclusively classified processed meat as carcinogenic to humans (Group 1), indicating a direct link between consumption of these products and an increased risk of developing cancer. Moreover, the IARC report also classified red meat as potentially carcinogenic to humans (Group 2A), suggesting a slightly lower but still significant level of risk associated with its consumption. The assessment of red meat's carcinogenic potential was informed by a substantial body of scientific literature and observational studies, indicating a credible but not yet definitive link between red meat intake and certain types of cancer. It is worth noting that while the evidence for red meat's carcinogenicity is less pronounced than that for processed meat, the IARC's classification underscores the importance of prudently evaluating and moderating the consumption of both processed and red meats as part of a healthy dietary regimen (Group 2A). Therefore, in the choice of meat, we should eat more white meat, eat red meat in moderation and as far as possible, do not eat fat meat and eat less or do not eat smoked meat, bacon, and other processed meat products.

In addition to daily diet, some nutraceuticals that have been clinically reported to be in use are also worthy of our attention. For example, resveratrol (RES), curcumin (CUR),^267^ and so on. Some studies have reported that RES and CUR can activate the expression of AMPK and SIRT1,^268^ which are important antiaging signaling pathways that also play a role in inhibiting tumor development. RES and CUR^269^ also inhibit signaling pathways such as WNT/β‐catenin, PI3K/Akt/mTOR, and RAS/MEK/ERK^267^ as well as regulate microRNA expression thereby affecting apoptosis, senescence, and the development of diseases such as cancer. In conclusion, some natural compounds and nutrients in the diet may exert antiaging and anticancer effects through multiple molecular mechanisms. Reasonable dietary habits may help prevent and delay the onset of these diseases.

## 9 SUMMARY AND OUTLOOK

By analyzing the number of cancer incidence and deaths in different age groups, we determined that aging can promote tumorigenesis. Based on this conclusion we wanted to explore the relationship at the level of molecular mechanisms. It is clear that the relationship between aging and tumors is intricate. On the one hand, an environment of intracellular ecological dysregulation and inflammation results in genomic instability, epigenetic changes, loss of protein homeostasis, mitochondrial dysfunction, and altered intercellular communication in senescent cells. These factors contribute to the proliferation and malignant advancement of cancer cells. On the flip side, tumor cells need to overcome the antagonistic hallmarks of senescence, telomere shortening, and stem cell depletion, in order to maintain the infinite proliferation and phenotypic plasticity of tumor cells.

Regarding the metabolic relationship between senescence and tumors, some researchers have suggested that senescence‐associated nutrient sensing dysregulation may lay the foundation for metabolic reprogramming in tumors. However, in fact, there is a contradiction between senescent cell metabolism and tumor cell metabolism, which needs to be analyzed in terms of specific metabolic modalities. Current findings suggest that glycolysis inhibits senescence in nontumor cells, but in tumor cells we have a different view. We know that tumors optimize energy supply through metabolic reprogramming, and that senescent cells, which use glucose as their primary energy source, similarly undergo metabolic changes. This led us to focus on exploring sugar metabolism in senescence and tumors. In tumor cells, senescence can generate ROS, leading to oxidative stress in tumor cells, and this oxidative stress environment helps tumor cells shift to aerobic glycolysis, thus promoting tumor development; in addition, senescent cells exhibit PDK4‐dependent aerobic glycolysis, which promotes an increase in intracellular lactic acid production, and a large amount of lactic acid formed generates ROS, which ultimately contributes to senescence of peripheral cells and even the tissue system and promote tumor proliferation. Similarly, it has been shown that senescence promotes tumor cell growth and proliferation through lipid metabolism, and lipid metabolism in the tumor microenvironment similarly affects cellular senescence and thus immune resistance to tumors; moreover, knockdown of HNF4α or SAA enzymes impairs hepatic SAA metabolism, promotes epithelial–mesenchymal transition, and promotes malignant tumor progression. Structural and functional changes in immune system components in senescent cells, amino acid metabolism, and other pathways regulate adaptive and innate immune cells with altered hair differentiation and response, increasing susceptibility to disease and thus promoting tumorigenesis. In summary, we conclude that senescent cells can spontaneously alter their metabolism like tumor cells, and that senescence promotes aerobic glycolysis, abnormal lipid metabolism, and amino acid metabolism in tumor cells, which in turn promotes the progression of malignant tumors.

We also summarized the signaling pathways involved in tumor aerobic glycolysis as well as in glycolysis in senescent cells and found that some signaling pathways in tumor cells also play a role in cellular senescence, such as the PI3K–AKT–mTOR pathway and the Ras signaling pathway, and can promote glycolysis in tumor cells, leading to increased tumor cell proliferation. In tumor cells, activation of the PI3K–AKT–mTOR pathway^60^ and the Ras signaling pathway can stimulate glycolysis, thereby enhancing the proliferation of tumor cells. We focused on the PI3K–AKT–mTOR signaling pathway because it plays a key role in cellular metabolic regulation and energy production. Activation of PI3K leads to cellular metabolic dysfunction and mitochondrial dysfunction that causes cellular senescence and promotes tumor progression. The upregulation of the YAP/TAZ activity in the Hippo pathway stimulates the transcriptional activation of HK2, which is pivotal in driving the glycolytic flux within cells promoting glycolysis. But YAP/TAZ activity is significantly downregulated in senescent cells, so the relationship between YAP/TAZ–HK2 regulation in senescent cells remains to be investigated. It is worth noting that cellular senescence leads to the generation of large amounts of ROS in the mitochondria, and the DNA damage caused by ROS can activate the classical oncogenic pathway P53 through p38/MAPK to play the role of oncogenicity; on the one hand, one of the markers of senescence SASP promotes the process of cellular senescence and inhibits tumorigenesis; on the other hand, SASP can also stimulate inflammation‐related responses and promote tumorigenesis and development. On the other hand, SASP can stimulate inflammation‐related responses and promote tumor development. This also proves the bidirectional role of senescence in tumor progression. Similarly, we have summarized the signaling pathways associated with abnormal lipid metabolism in tumor cells, such as NF‐κB, STAT3, Hippo, Wnt/β‐catenin, and Notch signaling pathways, which can all alter different aspects of lipid metabolism in tumor cells and increase lipid synthesis in tumor cells, thereby promoting tumor progression. It is noteworthy that senescent CAF have been found to enhance the formation of peritumoral tumors in GC through the JAK/STAT3 signaling pathway in the tumor microenvironment, which confirms our suspicion that senescence promotes the reprogramming of lipid metabolism during tumorigenesis and progression. In amino acid metabolism in tumors, we focus on amino acid classification and the specific biological roles of different amino acids in tumor cells. We found that glutamine is second only to glucose in importance in cancer, providing nitrogen and carbon to cancer cells and participating in a range of pathways including energy production, macromolecular synthesis, and signaling. Glutamate is converted to glutamine in large amounts during aging, causing metabolic disorders. It should be emphasized that both PI3K–mTOR pathway and AMPK pathway play a role in both tumor cells and senescent cells, and AMPK is activated in senescent cells, which participates in the regulation of cellular metabolism, affects intracellular amino acid levels, and alters the energy homeostasis of tumor cells, which in turn promotes their survival.

Since mitochondria are crucial in metabolism and one of the characteristics of senescence is mitochondrial dysfunction, we speculate that mitochondrial dysfunction is an important structural basis for cellular senescence and hence tumorigenesis. This is mainly achieved through mechanisms that induce oxidative stress, accumulation of mtDNA mutations, and effects on cell proliferation and growth. Factors contributing to mitochondrial dysfunction include defects in TCA cycle enzymes, mtDNA mutations,^180^ electron transport chain, oxidative stress, and aberrant expression of oncogenes and oncogenes. In particular, mutations in mtDNA in cancerous cells result in a rise in free radicals and higher levels of ROS within the tumor cells, which stimulate the proliferative and invasive properties of tumor cells. Mutant KRAS overexpression causes oxidative stress in mitochondria and oncogenic KRAS‐expressing cells show increased glycolytic activity, which promotes malignant tumor progression. In addition, the oncogene c‐Myc also induced mitochondrial oxidative stress and upregulated the NF‐κB signaling pathway, promoting tumorigenesis. The same lipid metabolism and amino acid metabolism occur on mitochondria, and abnormal amino acid metabolism is caused by mitochondrial translational malfunction and impaired TCA cycling in senescent cells. Amino acids require specific transporters to facilitate their role on mitochondria, but senescent cells cause mutations in their transporter SLC1A5, promoting tumor metabolic reprogramming. The lipid metabolism‐related protein TRPC3 remodels Ca^2+^ release leading to mitochondrial dysfunction and promotes tumorigenic effects in senescent cells. In addition, peroxisomes, the main site of lipid metabolism, play an important role in cellular lipid metabolism and the degradation of ROS, but their dysfunction also promotes tumorigenesis due to the decline of peroxisomal activity with increasing age. We thus conclude that mitochondrial dysfunction in senescent cells leads to increased intracellular ROS or promotes glycolysis, lipid metabolism, and amino acid metabolism, which in turn leads to tumor formation. We also summarize the drug therapeutic trials and related nanomaterials that can be retrieved to target aging and tumor‐related signaling pathways, with the research focusing on the PI3K/mTOR, AMPK, and Notch signaling pathway. In conclusion, we suggest that in our daily life, we need to optimize the composition and intake of our diet, and supplement some plant‐derived nutrients appropriately to achieve the goal of improving their health and realizing healthy aging.

The mechanisms of tumor and aging as well as tumor metabolism and aging metabolism still need to be studied and explored in depth, focusing on the aging phenotypes in tumor cells and further delving into the intricate molecular mechanisms underlying the aging process is crucial in elucidating its multifaceted role in driving tumor progression. With the progress of science, we believe that one day in the future we can lift the fog shrouding aging and tumor and overcome the difficult problem of healthy aging.

## AUTHOR CONTRIBUTIONS

*Conceptualization, investigation, data curation, writing—original draft, writing—review, and editing*: Yuzhu Zhang. *Data curation, software*: Jiaxi Tang and Can Jiang. *Review*: Hanxi Yi and Guang Shu. *Corresponding, supervision, project administration, funding acquisition, review, and editing*: Gang Yin. *Corresponding, project administration, funding acquisition, review, and editing*: Maonan Wang. All authors have read and approved the final manuscript.

## CONFLICT OF INTEREST STATEMENT

The authors declare no conflicts of interest.

## ETHICS STATEMENT

Not applicable.

## Data Availability

Not applicable.
